# A fine mapping of single nucleotide variants and haplotype analysis of *IL13* gene in patients with *Leishmania guyanensis*-cutaneous leishmaniasis and plasma cytokines IL-4, IL-5, and IL-13

**DOI:** 10.3389/fimmu.2023.1232488

**Published:** 2023-10-16

**Authors:** José do Espírito Santo Junior, Josué Lacerda de Souza, Lener Santos da Silva, Cilana Chagas da Silva, Tuanny Arruda do Nascimento, Mara Lúcia Gomes de Souza, Alyne Farias da Cunha, Jacqueline da Silva Batista, José Pereira de Moura Neto, Marcus Vinitius de Farias Guerra, Rajendranath Ramasawmy

**Affiliations:** ^1^ Programa de Pós-Graduação em Imunologia Básica e Aplicada, Instituto de Ciências Biológicas, Universidade Federal do Amazonas, Manaus, Amazonas, Brazil; ^2^ Faculdade de Medicina Nilton Lins, Universidade Nilton Lins, Manaus, Brazil; ^3^ Programa de Pós-Graduação em Biodiversidade e Biotecnologia da Amazonia Legal (Rede Bionorte), Universidade do Estado do Amazonas, Manaus, Brazil; ^4^ Fundação de Medicina Tropical Doutor Heitor Vieira Dourado, Manaus, Brazil; ^5^ Programa de Pós-Graduação em Medicina Tropical, Universidade do Estado do Amazonas, Manaus, Brazil; ^6^ Instituto Nacional de Pesquisa da Amazônia, Manaus, Brazil; ^7^ Faculdade de Ciência Farmacêuticas, Universidade Federal do Amazonas, Manaus, Brazil; ^8^ Genomic Health Surveillance Network: Optimization of Assistance and Research in The State of Amazonas – REGESAM, Manaus, Amazonas, Brazil

**Keywords:** *Leishmania guyanensis*, IL-13, single nucleotide variants, cutaneous leishmaniasis, susceptibility

## Abstract

**Introduction:**

Leishmaniasis continues to pose a substantial health burden in 97 countries worldwide. The progression and outcome of *Leishmania* infection are influenced by various factors, including the cytokine milieu, the skin microbiota at the infection site, the specific *Leishmania* species involved, the genetic background of the host, and the parasite load. In endemic regions to leishmaniasis, only a fraction of individuals infected actually develops the disease. Overexpression of IL-13 in naturally resistant C57BL/6 mice renders them susceptible to *L. major* infection. Haplotypes constructed from several single nucleotide variant (SNV) along a chromosome fragment may provide insight into any SNV near the fragment that may be genuinely associated with a phenotype in genetic association studies.

**Methods:**

We investigated nine SNVs (SNV1rs1881457A>C, SNV2rs1295687C>G, SNV3rs2069744C>T, SNV4rs2069747C>T, SNV5rs20541A>G, SNV6rs1295685A>G, SNV7rs848A>C, SNV8rs2069750G >C, and SNV9rs847T>C) spanning the entire *IL13* gene in patients with *L. guyanensis* cutaneous leishmaniasis (*Lg*-CL).

**Results:**

Our analysis did not reveal any significant association between the SNVs and susceptibility/protection against *Lg*-CL development. However, haplotype analysis, excluding SNV4rs2069747 and SNV8rs2069750 due to low minor allele frequency, revealed that carriers of the haplotype CCCTAAC had a 93% reduced likelihood developing Lg-CL. Similarly, the haplotypes ACCCGCT (ORadj=0.02 [95% CI 0.00–0.07]; *p*-value, 6.0×10^−19^) and AGCTAAC (ORadj=0.00[95% CI 0.00–0.00]; *p*-value 2.7×10^−12^) appeared to provide protection against the development of *Lg*-CL. Conversely, carriers of haplotype ACCTGCC have 190% increased likelihood of developing *Lg*-CL (ORadj=2.9 [95%CI 1.68–5.2]; *p*-value, 2.5×10^−6^). Similarly, haplotype ACCCAAT (ORadj=2.7 [95%CI 1.5–4.7]; *p*-value, 3.2×10^−5^) and haplotype AGCCGCC are associated with susceptibility to the development of *Lg*-CL (ORadj=1.7[95%CI 1.04–2.8]; *p*-value, 0.01). In our investigation, we also found a correlation between the genotypes of rs2069744, rs20541, rs1295685, rs847, and rs848 and plasma IL-5 levels among *Lg*-Cl patients. Furthermore, rs20541 showed a correlation with plasma IL-13 levels among *Lg*-Cl patients, while rs2069744 and rs848 showed a correlation with plasma IL-4 levels among the same group.

**Conclusions:**

Overall, our study identifies three haplotypes of *IL13* associated with resistance to disease development and three haplotypes linked to susceptibility. These findings suggest the possibility of a variant outside the gene region that may contribute, in conjunction with other genes, to differences in susceptibility and partially to the pathology.

## 1 Introduction

*Leishmania* is an obligate intracellular vector-borne protozoan parasite transmitted to humans through the bite of infected female sandflies. In the old world, the transmission is primarily by the *Phlebotomus* spp., while in the new world, it is mainly done by *Lutzomyia* spp. Leishmaniasis is an endemic disease in 97 countries worldwide, putting nearly one billion people at risk of *Leishmania* infection (1). It is a neglected disease that affects approximately 12 million individuals in tropical and subtropical countries, ranking as the ninth largest infectious disease burden according to the World Health Organization (1). Leishmaniasis causes approximately 2.4 million disability-adjusted life years lost (TDR Disease watch focus) and is the second highest cause of mortality and the fourth leading cause of morbidity among tropical diseases (2).

Leishmaniasis infection causes a wide spectrum of clinical manifestations depending on the *Leishmania* species. The clinical outcomes may range from asymptomatic self-healing lesions to cutaneous leishmaniasis (CL), diffuse CL, and disseminated CL, and severe mucocutaneous leishmaniasis (ML) in cases of tegumentary leishmaniasis. Additionally, it can lead to a complicated form known as visceral leishmaniasis (VL), which, if left untreated, can be fatal. Cutaneous leishmaniasis (CL), the most common form of the disease found in more than 88 countries, affects approximately 0.7 to 1.2 million individuals annually (3) and causes 41,700 DALYs (4).

The development of T helper 1 (Th1) response and the production of proinflammatory cytokines (IL-12, IL-2, TNF-α, IFN-γ, and IL-1β) during early *Leishmania* infection are beneficial to the host, as they lead to the activation of macrophages and parasite killing. In contrast, a Th2 response (IL-4, IL-5, and IL-13) promotes parasite persistence and disease development (5, 6). IFN-γ activates resting macrophages through the classical activation pathway, leading to their transformation into M1 macrophages. This activation inhibits pathogens multiplication and facilitates killing through the production of nitric oxide, reactive oxygen species, and lysosomal enzymes (7). In contrast, IL-4 and IL-13 activate resting macrophages to become alternative activated macrophages, also known as M2 macrophages, which exhibit anti-inflammatory properties (7). Studies conducted on animal models of leishmaniasis have suggested that achieving cure in leishmaniasis requires finely regulated cellular immune response and a delicate balance between pro- and anti-inflammatory cytokines (8, 9).

Murine experimental models have demonstrated the significance of IL-13 in leishmaniasis. Overexpression of IL-13 in C57BL/6 mice, which are typically resistant to *Leishmania major* infection, renders them susceptible to infection, irrespective of IL-4 expression (10). Conversely, BALB/c mice, known to be susceptible to *L. major* infection, exhibit resistance when the *IL13* gene is deleted (10). Exogenous IL-13 has been demonstrated to exert a hypoalgesic effect on mice infected with *L. major* and exacerbate the course of infection (11). Furthermore, the administration of exogenous IL-13 has been shown to increase the parasite burden and significantly decrease IFN-γ levels (12). The absence of IL-13 in mice infected with *L. amazonensis* significantly reduced paw swelling at the site of infection (13). Genetic mapping studies conducted in murine models have identified a genomic region that encompasses the *IL4* and *IL13* genes as a susceptibility region for leishmaniasis (14–16). In human, *IL4* and *IL13* are located on the long arm of chromosome 5 (5q.31–33).

At the genomic nucleotide sequence level, humans share 99.5% identity, while the remaining 0.5% sequence difference contributes, in conjunction with epigenetic modifications, to our phenotypic diversity. The 0.5% genomic nucleotide sequence difference is due to the presence of short and variable number tandem repeats, insertion or deletion polymorphisms, and single-nucleotide variants (SNPs) (17, 18). Among SNVs, the change from adenine to guanine and cytosine to thymine, or vice versa, is termed transitions, while the change from adenine to cytosine or thymine, and guanine to cytosine or thymine, is called transversions. In the human genome, an SNV with an allele frequency >1% occurs approximately every 100 to 300 base pairs, resulting in approximately 10 million SNVs within the human genome (18–20). Genome-wide association studies (GWAS) have revealed many specific SNVs linked to phenotypes or diseases (21).

SNVs present within the promoter region of a gene can affect mRNA expression, while SNVs within the gene may influence mRNA splicing, stability, or translation. A non-synonymous SNV leads to an amino acid change that can affect protein activity, while a synonymous SNV may lead to changes in translation rates or mRNA half-life. An SNV that introduces a premature stop codon results in a truncated protein (22, 23). A set of linked SNV alleles from a specific fragment of a chromosome that is transmitted as a block or tends to always occur together, as calculated by linkage disequilibrium (LD), is termed a haplotype. Haplotype analysis is interesting, as it might also provide insight into any SNV near the genetic fragment studied that may be genuinely associated with a phenotype in genetic association studies (24).

Genetic variations in *IL13* have been extensively investigated in the context of allergy and asthma and schistosomiasis. Particularly, the SNV rs1800925 located in the promoter region (−1112 C>T, with numbering relative to the *IL13* open reading frame ATG), and SNV rs20541, a non-synonymous variant situated in the fourth exon of the gene, have been studied (25). Additionally, studies have been conducted in schistosomiasis (26–30). However, research on genetic variations of *IL13* in protozoan infectious diseases remains limited. One study revealed an association between variant rs2069744 and *Plasmodium falciparum* malaria prevalence in northeast Tanzania (31). Furthermore, two studies conducted in Thailand indicated associations between SNVs rs1800925 (IL13 −1055T>C) (32) and rs1881457 (33) and severe malaria caused by *P. falciparum*. As of now, there exists only one study focused on the genetic determinants of *IL13* in the context of leishmaniasis. This study explored several candidate genes on chromosome 5q23.3-q31.1, which is in the proximity to the cluster of type 2 cytokines (Il-4, IL-5, and IL-13). The study investigated visceral leishmaniasis caused by *L. chagasi* and excluded the SNV rs848 located in the 3′ untranslated region (UTR) of *IL13*, due to its lack of polymorphism in the study population. However, an association was reported between the SNV rs2070874 of *IL4* and DTH^−^ phenotype in the Northeast of Brazil (34).

The outcome of *Leishmania* infection and the progression of disease development depend on various factors, including the cytokine milieu (35), the skin microbiota at the site of infection, the specific *Leishmania* spp., the genetic background of the host (36), and the parasite load (37). IL-13 has been observed in the majority of biopsy specimens taken from patients with *Lg*-Cl (38). In this manuscript, we performed a fine mapping of single nucleotide variants (SNVs) covering the whole gene of *IL13* to identify whether variants of *IL13* are associated with protection/susceptibility to *Lg*-CL or correlated with plasma cytokines IL-13, IL-4, and IL-5. We found that SNVs of *IL13* are not associated with protection/susceptibility to *Lg*-CL.

## 2 Materials and methods

### 2.1 Study population

The study involved 1,714 unrelated individuals (855 patients with *Lg-*CL and 859 healthy individuals (HCs) from the same endemic region of CL caused by *L. guyanensis*, as previously described (39–41). The research was conducted at the Fundação de Medicina Tropical Dr. Heitor Vieira Dourado (FMT-HVD), which serves as the reference center for tropical diseases. All participants were residents of the perirural area of Manaus, the capital city of the Amazonas state, Brazil. Specifically, they were from communities such as Pau-Rosa, Cooperativa, Água-Branca, Leão, and Brasileirinho, situated near BR-174 and AM-010, which have become endemic areas for *L. guyanensis* infection due to human encroachment. The population represents an admixture of Native American (50%–60%), European (40%–50%), and African (approximately 10%) ancestries (42). Patients were diagnosed with CL for the first time and presented with six or fewer lesions, with the majority having only one lesion. All patients had active CL. The healthy controls (HCs) were not stratified as asymptomatic, as we did not perform the delayed hypersensitivity test to *Leishmania* antigens. All the patients and the HCs tested negative for HIV and had no history of cardiac, renal, or diabetes disease. These healthy individuals underwent physical examinations by physicians to exclude any doubts regarding their history of leishmaniasis. They share the same socio-epidemiological environment and have been living in the endemic area for more than 5 years. Most the participants in this study are agricultural or farm workers. All participants are unrelated individuals. This case–control study compares unrelated patients with *Lg*-CL to healthy unrelated individuals. The study followed the guidelines for strengthening the reporting of genetic association studies (STREGA).

### 2.2 Ethical statements

This study adhered to the principles outlined in the Declaration of Helsinki and received approval from the Research Ethics Committee of the Fundação de Medicina Tropical Dr. Heitor Vieira Dourado under the file number CAAE:09995212.0.0000.0005. Written informed consent was obtained from all participants for the collection of biological samples and subsequent analysis. In the case of participants under the age 18, written informed consent was provided by the parent/guardian for the collection of biological samples and subsequent analysis.

### 2.3 Sample size calculations

The calculation for sample size for a case–control study investigating the immunogenetics of *Lg*-Cl has been previously described (43). In summary, the effective sample size was determined, using the Genetic Power calculator of Harvard University (http://pngu.mgh.harvard.edu/~purcell/gpc) considering multiple gene inputs for a trait. The calculation was based on several assumptions, including a minor allele frequency of 5%, disease prevalence of 5%, complete linkage disequilibrium between the marker and the trait, a case–control ratio of 1, a type 1 error rate of 5%, and an odds ratio of 1.5 and 2.0 for heterozygosity and homozygosity, respectively. To achieve 80% power, the genetic allelic model indicated that a sample size of 789 for cases and 789 for controls would be required.

### 2.4 Collection of biological samples and DNA isolation

Five milliliters of peripheral blood was obtained from each participant through venipuncture and collected into Vacutainers containing EDTA (Becton Dickinson, Sao Paulo, Brazil). The blood samples were used for both genomic DNA isolation, which was performed using the proteinase K salting-out method (44) and measurement of plasma circulating cytokines.

### 2.5 Identification of *Leishmania* species

Prior to collecting biopsy specimens from the skin ulcer lesion of all the patients with CL to identify the *Leishmania* sp., the presence of the parasite was confirmed by examining Giemsa-stained lesion scarifications under a microscope. For all patients with CL, DNA extracted from the biopsy specimens were subjected to *Leishmania vianna*-specific PCR with discrimination between *L. braziliensis* and *L. guyanensis* following previously established protocols (45, 46). To identify the *Leishmania* sp., direct nucleotide sequencing of a fragment of the HSP70 gene (233bp) and mini exon genes (227 bp) was performed (47). This sequencing was conducted using the kit BigDye Terminator v3.1 Cycle Sequencing (Thermo Fisher, MA, USA) following the manufacturer’s recommended protocols.

### 2.6 SNVs selection and genotyping

**Figure 1:**
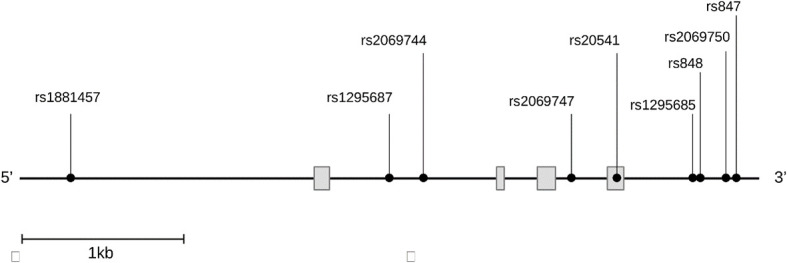
Distribution of the nine single nucleotide variants studied on the gene *IL13. IL13* gene figure displaying the genomic regions for each variant, rs1881457 (promoter), rs1295687 and rs2069744 (intron 1), rs2069747 (intron2), rs20541 (exon4), rs1295685, rs848, rs2069750, and rs847 (3′ untranslated region).

*IL13* gene harbors numerous SNVs with complex patterns of linkage disequilibrium (LD) in different populations. Nine SNVs spanning the whole gene of *IL13* were investigated as shown in **Figure 1** out of many SNVs present on the gene. The SNVs were selected from the public database of the HapMap Project SNPinfo Web Server (https://snpinfo.niehs.nih.gov) based on their minor allele frequencies of ≥5% with function prediction. These SNVs are tag SNP chosen from different populations according to the LD TAG SNP selection in linkage loci. The SNV rs1881457 A>C is situated in the promoter region, which is predicted to have binding sites for several transcription factors. Additionally, the SNVs rs1295687 C > G and rs2069744 C > T within the intron I also correspond to transcription factor binding sites. The rs20541 A>G is a missense mutation located in exon 4. The four SNVs (rs1295685 A>G, rs848 A>C, rs2069750 G >C, and rs847 T>C in the 3′ untranslated region (3′UTR) are likely to bind several miRNAs (https://snpinfo.niehs.nih.gov). These four SNVs in the 3′UTR also interact with the promoter of the long non-coding RNA, T helper type 2 locus control region associated RNA (*TH2LCRR*). Among the SNVs, the SNVs rs1295687 C > G, rs2069744 C > T, and rs2069747C>T were genotyped using PCR restriction fragment length polymorphism (PCR-RFLP), while the remaining SNVs were analyzed by direct nucleotide sequencing.

### 2.7 Alleles discrimination of the single nucleotide variants of *IL13* gene

Nine SNVs were selected for the study. The primers for polymerase chain reaction (PCR), amplification cycling conditions, and the size of the PCR products are listed in **Supplementary Table S1**. Primers were designed from the database Reference Sequence: NG_012090.1 from NCBI. Specifically, PCR was conducted in a final volume of 25 µL comprising the optimal concentration of MgCl_2_ in mmol/L, 0.2 pmol/L each of forward and reverse primer, 40 µmol/L of each dNTP, 50 ng of DNA, 1 U of *Taq polymerase* and 2.5 µL of 10× Taq polymerase buffer containing 500 mmol/L KCl and 100 mmol/L Tris-HCL. PCR reactions were conducted using the Applied Biosystem Veriti Thermal Cycler.

The genotypes of SNVs rs1295687, rs2069744, and rs2069747 were determined using PCR-RFLP. The sizes of the generated PCR were 279 base pair (bp) for rs1295687, 137 bp for rs2069744, and191 bp for rs2069747. A volume of 10 µL of the PCR products was digested with the respective restriction enzymes: *Dde*I, *Hinf*I, and *Nco*I. In the presence of the C allele of rs1295687, the enzyme *Dde*I cleaves the 297-bp fragment into 187 bp and 92 bp, while in the presence of G allele, into 142 bp, 92 bp, and 45 bp. Similarly, for rs2069744, *Hinf*I cleaves the137-bp fragment into 114 bp and 23 bp when the C allele was present, and it remained uncut when the T allele was present. Finally, for the rs1295687, *Nco*I cuts the 191-bp fragment into 121 bp and 70 bp in the presence of the C allele and uncut for the T allele.

For the SNVs located in the 3′ untranslated region (3′UTR), we designed a pair of primers that flank four SNVs: rs1295685 A>G, rs848 A>C, rs2069750 G >C, and rs847 T>C, which generated a 459-bp PCR fragment. The sizes of the PCR products of the SNV rs1881457 A>C in the promoter region and the rs20541 A>G in exon were 275 bp and 210 bp, respectively. After PCR amplifications, the PCR products were purified using a 20% PEG purification method following the protocols described elsewhere (48, 49). The purified amplicons were subjected to sequencing using either the forward or reverse primers of each fragment. The sequencing reactions were performed using the BigDye Terminator v3.1 Cycle Sequencing Kit (Thermo Fisher, MA, USA) according to the manufacturer’s protocol in the Applied Biosystem Veriti Thermal Cycler. The resulting sequencing products were analyzed using the ABI 3130XL automatic DNA Sequencer with POP-7 as the sequencing polymer. Nucleotides reading were obtained using the Sequencing Analysis software (Applied Biosystems, v5.3.1), and only high-quality sequences were utilized for SNP analyses.

### 2.8 Assay of circulating plasma cytokines IL-4, IL-5, and IL-13 by Luminex

Plasma samples were kept frozen at −80°C until measurement of cytokine levels. IL-4, IL-5, and IL-13 cytokines levels in 5 μL of plasma were measured using the Human Cytokine Grp I Panel 27-Plex kit (Bio-Rad, USA) through a multiplex cytokine assay. The assay was conducted following the manufacturer’s instruction on the Bio-plex 200 Protein Array System (Luminex Corporation, USA).

### 2.9 Statistical analysis

Genotypes and alleles frequencies were calculated using gene counting method. Hardy–Weinberg equilibrium was assessed by comparing observed frequencies to expected frequencies of the genotypes. Statistical comparison of genotypes and alleles between patients with CL and HCs was conducted using logistic regression, which included odd ratio (OR) and 95% confidence interval (CI) with adjustment for sex and age. Different inheritance models (codominant, dominant, recessive, and overdominant models) were analyzed using R software version 4.3.1 with the SNPassoc package 2.1-0. The effects of genotypes on circulating plasma cytokine levels of IL-4, IL-5, and IL-13 were evaluated using the Generalized Linear Model (GLM) for quantitative traits in the R software of the SNPassoc package. Visualization of cytokine results was achieved using the ggplot2 package. The Akaike Information Criterion (AIC) was utilized to indicate the best model in both logistic regression and GLM. *Post-hoc* analysis following ANOVA was carried out using the package postHoc version 0.1.3 in R software (r-project.org) to compare the effect levels based on genotypes, and the *p*-values were adjusted by the Bejamini–Hochberg method. Linkage disequilibrium (LD) test and LD visualization were conducted using Haploview software 4.2. Haplotypes analysis was performed using the haplot.stats version 2.1-0.

## 3 Results

### 3.1 Characteristics of study population

Out of the 1,714 individuals included in the study, 855 were diagnosed with *Lg-*CL, while the remaining 859 individuals who had no history and scar of leishmaniasis were considered healthy controls (HCs). The HCs were selected from the same endemic area of the patients with *Lg*-CL and underwent thorough physical examinations by physicians to ensure the absence any previous history of leishmaniasis. Of note, only patients infected with *L. guyanensis* were included in the study after identification of the *Leishmania* species. They shared the same socio-epidemiological environment and had resided in the endemic area for over 5 years. All the participants were unrelated individuals, and most of them were agricultural or farm workers. All patients included in the study presented with active CL and had fewer than or equal to six ulcer skin lesions. Among the patients with *Lg*-CL, 639 were male with a mean age of (mean ± standard error of the mean) 34.29 ± 0.53 years, while the remaining 216 female participants had a mean age of 37.19 ± 1.05 years. In the HC group, there were 591 male participants, with a mean age of 42 ± 0.72 years and 268 female participants, with a mean age of 40 ± 1.04 years. All the participants were free of HIV, cardiac, renal, or diabetic diseases. Male HC participants were slightly older than male patients with *Lg*-CL (p< 0.0001). The characteristics of the study population is shown in **Table 1**.

**Table 1:** Demographic characteristics of study population.

	Cases (n =855)	HCs (n = 859)			*p*-value
	Male	Female	Male	Female	
**Sex**	639 (75%)	216 (25%)	591 (69%)	268 (31%)	
**Age (means) ± SEM**	34.29 ± 0.53	37.19 ± 1.05	41.81 ± 0.72	40.17 ± 1.04	<0.0001

HCs, healthy controls; SEM, standard error of mean.

χ^2^ and Mann–Whitney tests were used to compare sex and age differences; p-value < 0.05 are considered significant.

The minor allele frequencies (MAFs) of the nine SNVs among the patients with *Lg-*CL, healthy controls, and global MAF from the HapMap project are presented in **Table 2**. All the SNVs were in Hardy–Weinberg equilibrium. The genotype frequencies and statistical comparisons, based on different genetics models, between patients with *Lg-*CL and healthy controls, are provided in **Supplementary Table S2**. Importantly, none of the SNVs demonstrated any association with susceptibility or protection against the development of *Lg*-CL.

**Table 2:** Minor allele frequencies (MAFs) of the single nucleotide variants among the patients with *Lg*-CL, healthy controls, and global MAF from HapMap project.

Gene region	Markers	Alleles	Patients with CL (%)	Healthy controls (%)	Global MAF	*p*-value
**Promoter**	rs1881457	C	379 (22%)	359 (21%)	19%	n.s.
**Intron1**	rs1295687	G	287 (18%)	284 (18%)	17%	n.s.
**Intron1**	rs2069744	T	190 (11%)	215 (13%)	17%	n.s.
**Intron3**	rs2069747	T	26 (2%)	22 (1%)	5%	n.s.
**Exon4**	rs20541	T	620 (36%)	654 (38%)	23%	n.s.
**3′ UTR**	rs1295685	A	631 (36%)	648 (38%)	17%	n.s.
**3′ UTR**	rs848	A	720 (42%)	766 (45%)	37%	n.s.
**3′ UTR**	rs2069750	C	29 (2%)	48 (3%)	12%	n.s.
**3′ UTR**	rs847	T	650 (38%)	662 (39%)	19%	n.s.

Comparisons of IL13 variants with p-value adjusted by sex and age performed in R software. p-value < 0.05 are considered significant. n.s., not significant.

### 3.2 Linkage disequilibrium and haplotype analysis

**Figure 2:**
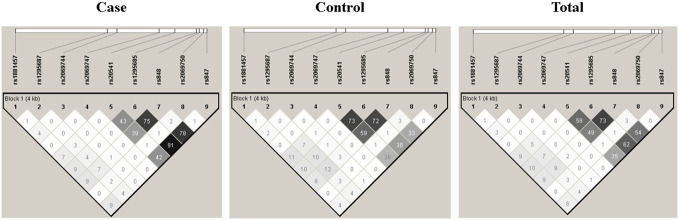
Linkage disequilibrium structure among the nine single nucleotide variants of the *IL13*.

The LD analysis was performed on the nine SNVs using the Haploview Software V4.2, and the results are presented in **Figure 2**. Among the SNVs, namely, rs20541, rs1295685, rs848, and rs847, a strong LD was observed, indicating association with each other. These SNVs form a distinct block in comparison to the remaining SNVs.

Haplotype analysis was conducted using the haplot.stats version 2.1-0 to assess the haplotype frequencies from the nine SNVs among the patients with *Lg*-CL and HCs. A total of 38 haplotypes were identified without applying a threshold of frequency. However, when frequency limit to 0.1% was set, a total of 14 haplotypes were observed. The haplotypes are presented in **Supplementary Table S3**.

Since the MAF of SNV4 (rs2069747) and SNV8 (rs2069750) were approximately 2%, we decided to exclude these SNVs from further analysis. Consequently, we recalculated the haplotype frequencies, and the updated results are as shown in **Table 3**. A total of 15 haplotypes was observed.

**Table 3:** Distribution of the haplotypes frequencies of the *IL13* gene among the patients with *Lg*-CL (cases) and healthy controls (HCs) as identified after excluding the single nucleotide variants rs2069747 and rs2069750.

H	Variants	Cases (%)	HCs (%)	*p*-value [OR (CI 95%)]	Adj *p*-value [OR (CI 95%)]
1	ACCCGCC	640 (3.8)	597 (35)	0.14 [1.1 (0.9–1.2)]	0.14 [1 (0.0–0.0)]
2	ACCTAAT	137 (8.1)	117 (7)	0.21 [1.2 (0.9–1.5)]	0.17 [1.06 (0.7–1.4)]
3	AGCTAAT	123 (7.3)	124 (7.4)	0.89 [0.9 (0.7–1.2)]	0.58 [0.76 (0.56–1.03)]
4	CCCTAAT	130 (7.7)	116 (6.9)	0.38 [1.1 (0.8–1.4)]	0.39 [1.06 (0.81–145)]
5	CCCCGCC	74 (4.4)	66 (4)	0.50 [1.1 (0.8–1.5)]	0.36 [1.18 (1.04–2.81)]
6	ACTCGCC	63 (3.7)	58 (3.5)	0.69 [1.1 (0.7–1.5)]	0.86 [1.06 (0.68–1.69)]
7	AGCCGCC	64 (3.8)	43 (2.6)	0.04 [1.5 (1–2.2)]	0.01 [1.71 (1.04–2.81)]
8	ACCTGCC	75 (4.4)	29 (1.8)	7.1e–6 [2.8 (1.8–4.3)]	2.52e–06 [2.94 (1.68–5.2)]
9	CCTTAAT	49 (2.9)	50 (3)	0.91 [1 (0.6–1.4)]	0.89 [1.18 (0.74–1.89)]
10	ACCCGCT	7 (0.4)	90 (5.4)	6.1e–18 [0.07 (0.03–0.15)]	6.0e–19 [0.02 (0.00–0.07)]
11	ACCCAAT	56 (3.3)	21 (1.3)	7.9e–5 [2.7 (1.6–4.4)]	3.20e–05 [2.66 (1.51–4.71)]
12	ACCCGAC	21 (1.2)	28 (1.7)	0.29 [0.7 (0.4–1.4)]	0.41 [0.66 (0.34–1.28)]
13	AGCTAAC	2 (0.1)	46 (2.7)	1.4e–10 [0.04 (0.01–0.15)]	2.74e–12 [6.01e–74 (0.00–0.00)]
14	CCCTAAC	3 (0.2)	38 (2.3)	3.3e–8 [0.07 (0.02–0.2)]	1.08e–10 [7.74e–29 (0.00–0.00)]
15	AGCCGAC	18 (1.1)	22 (1.3)	0.47 [0.8 (0.4–1.5)]	0.79 [0.82 (0.39–1.71)]

The haplotype consists of rs1881457, rs1295687, rs2069744, rs20541, rs1295685, rs848, and rs847.

Hap, haplotypes; HCs, healthy controls; OR, odds ratio; CI 95%, confidence interval; adj., sex and age adjusted. p-value < 0.05 are considered significant.

The distribution of haplotypes exhibits notable difference between the patients with *Lg*-Cl and HCs. Among the HCs, there is an increased occurrence of the haplotype ACCCGCT (5.4%), AGCTAAC (2.7%), and CCCTAAC (2.3%) when compared to the patients with *Lg*-CL [ACCCGCT (0.4%), AGCTAAC (0.1%), and H13 (0.2%)]. Statistical comparison between patients with *Lg*-CL indicates that bearers of the haplotype CCCTAAC may have 93% decreased likelihood of developing *Lg*-CL, and when adjusted for age and sex, the chance of developing the disease is almost null. Similarly, the haplotypes ACCCGCT (ORadj = 0.02 [95% CI, 0.00–0.07]; *p*-value, 6.0 × 10^−19^) and AGCTAAC (ORadj = 0.00 [95% CI, 0.00–0.00]; *p*-value 2.7 × 10^−12^) provide protection against the development of *Lg*-CL. By contrast, individuals bearing the haplotype ACCTGCC have 190% increased likelihood of developing *Lg*-CL (ORadj = 2.9 [95% CI, 1.68–5.2]; *p*-value, 2.5 × 10^−6^). Similarly, carriers of the haplotype ACCCAAT have 170% odds of developing the disease *Lg-CL* (ORadj = 2.7 [95% CI, 1.5–4.7]; *p*-value 3.2 × 10^−5^). Similarly, the haplotype AGCCGCC is associated with susceptibility to the development of *Lg*-CL (ORadj = 1.7 [95% CI, 1.04–2.8]; *p*-value 0.01).

### 3.3 Correlation of SNVs genotypes with plasma cytokines IL-13, IL-4, and IL-5

**Figure 3:**
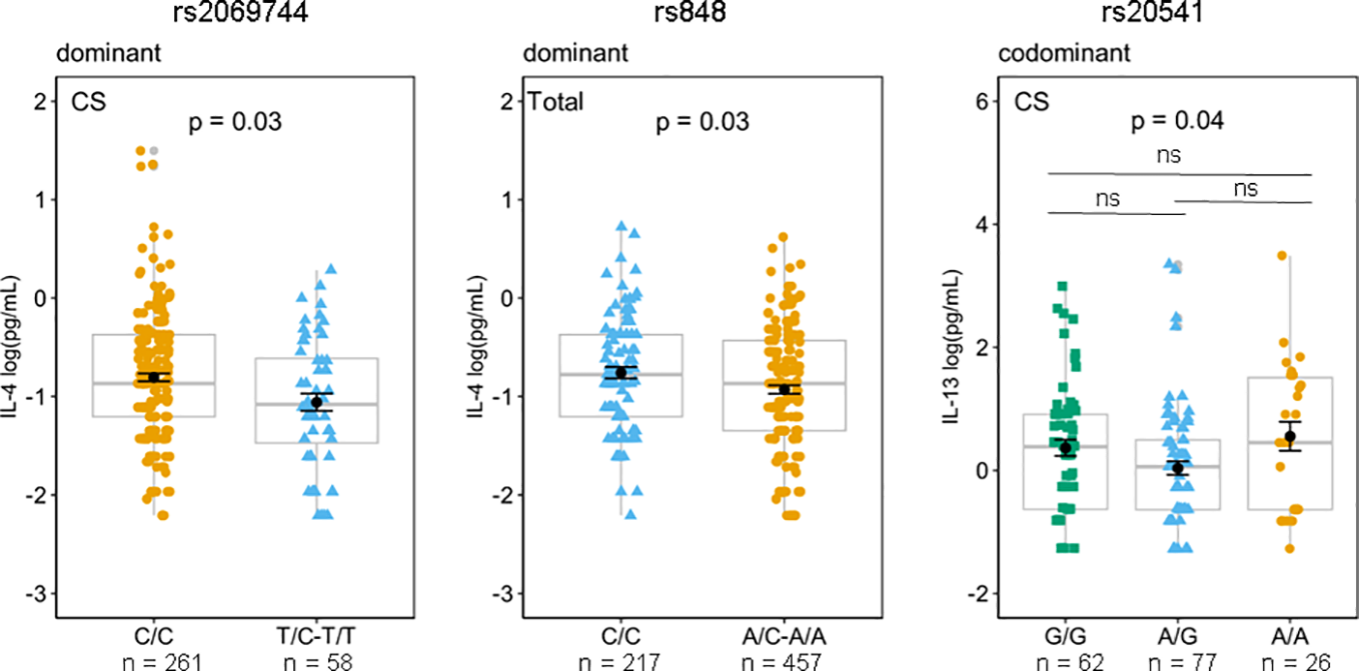
Effects of genotypes rs2069744 and rs848 on circulating plasma IL-4 based on a dominant genetic model while rs20541 on IL-13 in the codominant model. Mean of IL-13 concentrations were compared between the genotypes in patients with *Lg*-CL (CS), and in the Total (*Lg*-CL + HCs) by Generalized Linear Model (GLM) considering the inheritance models. P represents *p*-values adjusted for covariates age and sex. The means expressed in log (pg/mL) are represented by black dots, while the error bar is the standard error of the mean; boxplots in gray represent the median and quartiles and are used to show the distance between mean and median. *Post-hoc* analysis for rs20541/IL-13 using the postHoc package. ns, not significant.

Based on the findings from animal models of leishmaniasis that high levels of IL-13 render them susceptible to infection (10–13), we investigated whether any genotype of the SNVs studied may be related to high or low levels of plasma IL-13 to explain the susceptibility/protection to *Lg*-Cl. The mean and standard error of the mean of circulating plasma levels of IL-4, IL-5, and IL-13 according to the genotypes and based on different genetics models in pg/mL are given in **Supplementary Tables S4–S6**. The rs2069744, situated in the intronic region demonstrated solely a correlation with plasma IL-4 levels among the patients with *Lg*-Cl in a dominant genetic model. Likewise, rs848, situated in the 3′UTR, displayed a correlation with plasma IL-4 levels among the entire study population (including patients with *Lg*-Cl and HCs) in a dominant genetic model (**Figure 3**). The homozygous genotype CC of rs2069744 and of rs848 exhibited a correlation with elevated levels of IL-4. Notably, only rs20541 demonstrates a correlation with plasma IL-13, and this association is observed specifically in patients with *Lg*-CL (**Figure 3**). However, *post-hoc* analysis did not reveal any significant association, even though homozygous individuals with genotype AA exhibited a higher mean of 0.55log(pg/mL) compared to homozygous individuals with genotype GG which had a mean of 0.37 log(pg/mL) of IL-13.

**Figure 4:**
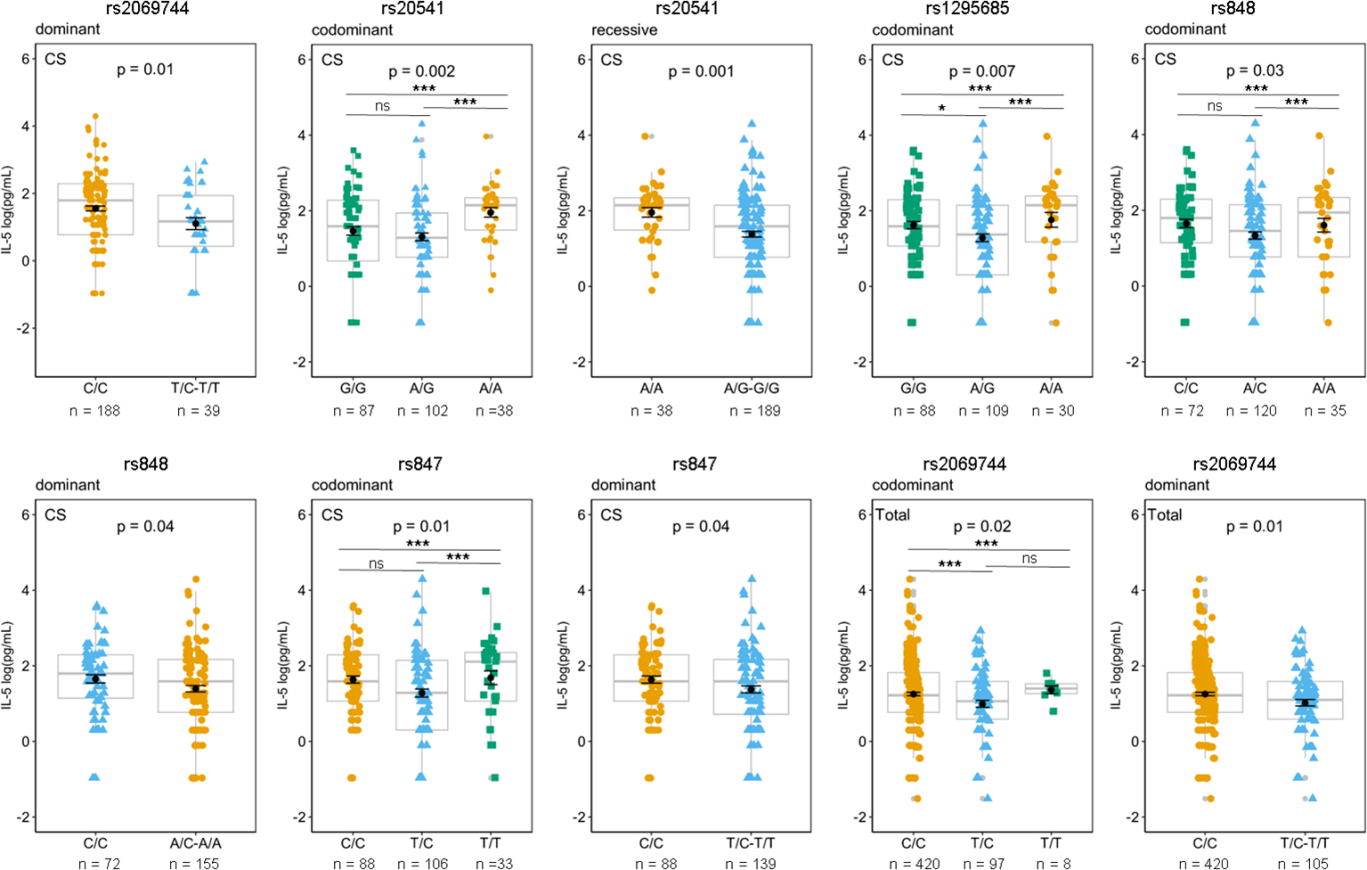
Effects of genotypes rs2069744, rs20541, rs129685, rs848, and rs847 on circulating plasma IL-5 based on different genetic model. The mean of IL-13 concentrations was compared between genotypes in patients with *Lg*-CL (CS), HCs (healthy controls), and Total (*Lg*-CL+HC) by Generalized Linear Model (GLM) considering the inheritance models. P represents *p*-values adjusted for covariates age and sex. The means expressed in log (pg/mL) are represented by black dots, while the error bar is the standard error of the mean; boxplots in gray represent the median and quartiles and are used to show the distance between mean and median. *Post-hoc* analysis for rs2069744, rs20541, rs1295685, rs848, and rs847 using the postHoc package. *p*-value <*0.05, **0.01, ***0.001. ns, not significant.

The association between specific genetic variants and plasma IL-5 levels among patients with *Lg*-CL is observed with several SNVs, including rs2069744, rs20541 (in the exonic region), and three SNVs in the 3′UTR, namely, rs1295685, rs848, and rs 847. These associations are illustrated in **Figure 4**. The homozygous genotype CC of rs2069744, AA of rs20541, AA of rs1295685, AA of rs848, and TT of rs847 are correlated with higher mean levels of IL-5.

*Post-hoc* analysis using the postHoc package was conducted for the variants rs20541, rs1295685, rs848, rs847, and rs2069744 in relation to plasma levels of IL-5. The results displayed significant association between IL-5 levels and their corresponding genotypes, as shown in **Figure 4**. For rs20541, individuals with the AA genotype exhibited a significantly higher mean plasma IL-5 levels 2.0 log (pg/mL) compared to those with the rs20541 GG genotype, which had a mean of 1.5 log (pg/mL) in patients with *Lg*-CL. This suggests that the A variant is associated with a higher level of IL-5.

Similarly, for rs1295685, the AA genotype was significantly associated with higher plasma IL-5 levels, with a mean of 1.8 log (pg/mL) compared to the GG genotype, which had a mean of 1.6 log (pg/mL) in patients with *Lg*-CL. This suggests that A variant is associated with higher levels of IL-5. Furthermore, the rs848 A variant was correlated with higher levels of IL-5.

For rs847, individuals with the homozygous TT genotype [1.68 log (pg/mL)] exhibited higher levels of IL-5 compared to those with CC genotype [1.64 log (pg/mL)] and TC genotype (1.25 log [pg/mL)] and TC genotype in patients with *Lg-*Cl, suggesting that the variant T is correlated with higher IL-5.

Similarly, for rs2069744, homozygotes with TT (1.37 log(pg/mL) is correlated with higher levels of IL-5 compared to homozygotes with the CC genotype (1.24 log(pg/mL) among the total subjects, suggesting that the variant T is correlated with higher level.

## 4 Discussion

The current cross-sectional study was designed to examine the genetic susceptibility by focusing on SNVs in the *IL13* gene in individuals with *Lg*-CL and to assess their impact on the regulation of circulating plasma cytokine IL-4, IL-5 and IL-13. IL-13 is a significant Th2 cytokine and shares important similarities with IL-4. Th1 and Th2 cytokines play a crucial role in the immunopathogenesis of CL, with Th1 cytokines associated with resistance and Th2 cytokines with susceptibility to infection.

We did not observe any association between the nine SNVs of *IL13* investigated in this study and susceptibility or protection to the development of *Lg*-CL. Conversely, studies conducted in *P. falciparum* malaria have demonstrated that the variant rs2069744 is associated with *P. falciparum* malaria prevalence (31), and the SNVs rs1800925 and rs1881457 are linked with severe malaria caused by *P. falciparum* (32, 33). However, our study revealed that the three haplotypes ACCTGCC, AGCCGCC, and ACCCAAT are associated with susceptibility to the development of *Lg*-CL, whereas the three haplotypes ACCCGCT, AGCTAAC, and CCCTAAC with protection. Naka et al. (2009) studied 82 SNVs within a 522-kb region on chromosome 5q31-33 and observed two haplotypes encompassing the entire *RAD* gene and the promoter of *IL13* bearing the C allele of rs188147 conferring protection to severe malaria (33). Interestingly, none of the susceptibility-associated haplotypes for *Lg*-Cl bear the C allele of rs188147.

Among the nine SNVs studied, the genotype of rs2069744, situated in the intronic region 1, showed correlation with circulating plasma cytokines IL-4 and IL-5 among the patients with *Lg*-CL and with IL-5 in the entire study population. The rs20541 is a non-synonymous variant (Arg130Gln) that results from the substitution of the amino acid arginine with glutamine at codon 130. The G allele yields arginine, while A allele yields glutamine. This variant has been associated with total IgE concentrations in several studies (50–55). Gln130 has been demonstrated to exhibit higher activity on primary effector cells involved in human allergic inflammation compared to the common Arg130. This finding led the authors to suggest that the increased allergic inflammation is likely due to enhanced IL-13-mediated Th2 effector functions, rather than Th2 differentiation (54). This gain of function variant has been demonstrated to be associated with increased *IFNG* gene expression in the peripheral blood (55). The replacement of Arg130 with 130Gln, located in the α-helix D of the IL-13 protein, is a region critical for its interaction with its receptors IL-4Rα1/IL-13Rα2. The 130Gln variant has been shown to display reduced affinity with IL-13Rα2, a decoy receptor, resulting in its decreased clearance (56). In this study, homozygous carriers with the AA (Gln130) genotype appear to exhibit a correlation with elevated plasma IL-5 and IL-13 levels. Indeed, Wang et al. also observed that the AA genotype is associated with higher plasma levels of IL-13 in patients with systemic lupus erythematous (57). Notably, this SNV rs20541 has been shown to exhibit weak LD with rs1800925 in the promoter region (−1112 C/T), which enhances *IL13* expression in human Th2 cells by creating a binding site for the transcription factor YY1 and relieving STAT6-mediated repression (58).

Regarding the SNV rs1881457 located in the promoter region (*IL13*-1512A>C), no association between IL-13 levels and its genotypes was observed. This SNV rs1881457 has been identified as a functional variant that enhances *IL13* expression by creating a binding site for the transcription factor Oct-1 (59). Oct-1 exhibits a preferential binding affinity for the C allele. Situated within a nuclease hypersensitive site, the rs1881457A>C has been demonstrated in vector-reported assays to upregulate the *IL13* proximal promoter in transiently transfected differentiated primary murine CD4+ Th2 cells, with the C allele displaying higher activity (59). Diverse findings have been reported by different research groups studying the SNV rs1800925 in the promoter region (−1112 C/T) and its association with IL-13 levels. One study observed an association between high levels of IL-13 and the CC genotypes in schistosomiasis-uninfected individuals in Zimbabwe (60), while another study indicated that TT genotypes are correlated with high IL-13 levels in *S. japonicum*-infected individuals (61). Another SNV rs2069739A>G, an intron variant of IL13, has also been linked with plasma levels of IL-13. Carriers of the allele A of SNV rs2069739 are associated with an increased risk of having low plasma concentrations of IL-13 (30, 60).

The SNVs rs1295685, rs848, and rs847 located in the 3′UTR region of the *IL13* gene have been demonstrated to interact with the promoter region and thereby regulating the expression of the long non-coding RNA T helper type 2 locus control region associated RNA (Th2LCRR) (62). Th2LCRR is transcribed as an antisense of the RAD50 gene (63). Th2LCRR is situated in the same chromosomal region of IL-4, IL-5, and IL-13 on chromosome 5 and maintains epigenetics effects on the promoters of IL-4, IL-5, and IL-13, thereby influencing the regulation of T-cell polarization (64). The SNVs rs1295685, rs848, and rs847 exhibit weak LD with rs20541 in our population, whereas they display a strong LD in European population (62). Li et al. (2022) demonstrated that haplotypes containing the GCC sequence of the 3′UTR of IL13, when inserted in plasmid pGL3 promoter, exhibited higher luciferase activity compared to the haplotypes with AAT sequence. Conversely, the haplotypes with ACC, GAC, and GCT sequence displayed very low activity. Additionally, the G allele of rs1295685 was found to be associated with higher expression of *TH2LCRR* compared to the A allele (62). Interestingly, our study showed two haplotypes bearing the GCC sequence of the three SNVs in the 3′UTR (ACCTGCC; OR_adj_ =2.9 [CI95%, 1.7–5.2)], *p*-value 2.52e−06 and AGCCGCC OR_adj_ =1.7 [CI 95% [1.0–2.8], *p*-value 0.01) are associated with susceptibility to the development of *Lg*-CL. Conversely, haplotype containing the GCT sequence of the 3′UTR revealed a significant association with protection against disease development (ACCCGCT; OR_adj_=0.02 [CI 95% [0.00–0.07]; *p*-value, 6×10^−19^). We established haplotypes using the only SNVs (rs1295685, rs848, and rs847) in the 3′UTR of *IL13*, and the distribution of these haplotypes is presented shown in **Supplementary Table S7**. Remarkably, the haplotype GCC (OR_adj_ = 1.8 [CI95%, 1.4–2.0]; *p*-value, 4.0×10^−7^) and the AAT haplotype (OR_adj_ = 1.4 [CI95%, 1.2–1.7]; *p*-value, 2.0×10^−4^) exhibited an association with susceptibility to the development of *Lg*-CL. In contrast, the haplotype AAC (OR_adj_ =0.05 [CI95%, 0.03–0.11]; *p*-value, 3.2 × 10^−28^) and the GCT haplotype (OR_adj_ = 0.04 [CI95%, 0.02–0.10]; *p*-value, 7.0 × 10^−24^) were associated with protection. *TH2LCRR* is known to regulate the expression of IL-4, IL-5, and IL-13 (65). It influences the expression of Th2 cytokine genes through histones modification, and there is a positive correlation between *TH2LCRR* and *IL4*, *IL5*, and *IL13* (63). *TH2LCRR* knockout mice show a significant reduction in Th2 cytokines (66). Indeed, numerous studies have underlined the significance of the chromosome 5 region, which harbors a cluster of immune response genes crucial for regulating both Type1 and Type 2 immune responses. This region has also been implicated in controlling the susceptibility to parasitic diseases (26, 67, 68). In this study, the SNVs rs1295685, rs848, and rs847 are correlated with plasma IL-5.

*L. major*-infected BALB/c mice display a persistent Th2 response with high levels of IL-4 and IL-13 in contrast to C57BL/6 mice and develop ulcerative skin lesions (69). Several studies have reported an accumulation of eosinophils at the infection site in murine models of CL (70–75). Eosinophils are known to produce IL-4 and IL-13, which can contribute to the maintenance of TH2 cells and subsequently may promote parasites persistence by deactivating M1 macrophages in susceptible individuals. In fact, a study conducted on genetically resistant C57BL/6 mice infected with *L. major* Seidman strain reported the deactivation of M1 macrophages by eosinophil-derived IL-4, rendering these mice susceptible to infection (71). In naturally resistant C57BL/6 mice infected with *L. major* Seidman strain, a progressive course of infection is observed despite the development of Th1 cells (76), while in mice with a specific deletion of IL-4 and IL-13 in eosinophils, clinical amelioration is shown, indicating the role of these cytokines in promoting disease progression (71).

Considering the findings from animal models of leishmaniasis (69–76), it is reasonable to propose that individuals harboring the haplotype associated with susceptibility to the development of *Lg*-Cl might display increased expression of *TH2LCRR.* This heightened expression could potentially result in sustained polarization of Th2 cells and the subsequent release of IL-4 and IL-13. Elevated levels of IL-4 may play a role in deactivating M1 macrophages, consequently hindering the efficient elimination of the *Leishmania* pathogen. This scenario could potentially contribute to disease progression and development.

In summary, the present study provides the first evidence in a large sample of patients with *Lg*-CL that individual SNVs of *IL13* are not associated with protection/susceptibility to *Lg*-CL. However, haplotypes associated with high Th2 cytokines levels are found to be associated with susceptibility to *Lg*-CL disease development. Our study identified three haplotypes of *IL13* linked to resistance and three haplotypes linked to susceptibility. These findings suggest the potential presence of a variant located outside the *IL13* gene region that may contribute to susceptibility with *Lg*-CL in conjunction with other genes that may be responsible for differences in susceptibility.

## Data availability statement

The original contributions presented in the study are included in the article/**Supplementary Material**. Further inquiries can be directed to the corresponding author.

## Ethics statement

The studies involving humans were approved by Research Ethics Committee of the Fundação de Medicina Tropical Doutor Heitor Vieira Dourado (FMT-HVD). The studies were conducted in accordance with the local legislation and institutional requirements. Written informed consent for participation in this study was provided by the participants’ legal guardians/next of kin.

## Author contributions

JJ, JS, and RR contributed for data curation. JJ, MG, and RR take responsibility for the integrity of the work as a whole, from inception to published article. JJ and RR were responsible for study design and conception and drafted the manuscript. JJ, JS, LS, CS, TN, MS, AC, JB, and JN were responsible for PCR and nucleotide sequencing. JJ, JS, MG, and RR collected and cleaned the data for formal analysis. JJ and JS were responsible for statistical analysis. JJ and RR interpreted the results and drafted the manuscript. MG and RR supervised the whole work. All authors revised the manuscript for important intellectual content. All authors contributed to the article and approved the submitted version.
