# Supplementary material for: A fine mapping of single nucleotide variants and haplotype analysis of IL13 gene in patients with Leishmania guyanensis-cutaneous leishmaniasis and plasma cytokines IL-4, IL-5, and IL-13
Source: Front Immunol. 2023 Oct 16;14:1232488. doi: 10.3389/fimmu.2023.1232488 (PMC10613733; doi:10.3389/fimmu.2023.1232488)
Supplement: Supplementary file 1 [file Table_1.docx]

Supplementary Material

**A fine mapping of single nucleotide variants of *IL13* gene in patients with *Leishmania* *guyanensis*-cutaneous leishmaniasis and plasma cytokines IL-4, IL-5 and IL-13**

José do Espírito Santo Junior^1,2^, Josué Lacerda de Souza ^2,3^, Lener Santos da Silva^3^, Cilana Chagas da Silva^4,5^, Tuanny Arruda do Nascimento^2,4^, Mara Lúcia Gomes de Souza^4^, Alyne Farias da Cunha^6^, Jacqueline da Silva Batista^6^, José Pereira de Moura Neto^7^, Marcus Vinitius de Farias Guerra^4,5^ and Rajendranath Ramasawmy^1,2,4,5,8^

^1^Programa de Pós-Graduação em Imunologia Básica e Aplicada, Instituto de Ciências Biológicas, Universidade Federal do Amazonas, Manaus, Amazonas, Brazil.

^2^Faculdade de Medicina Nilton Lins, Universidade Nilton Lins, Manaus, Brazil.

^3^Programa de Pós-Graduação em Biodiversidade e Biotecnologia da Amazonia Legal (Rede Bionorte), Universidade do Estado do Amazonas, Manaus, Brazil.

^4^Fundação de Medicina Tropical Doutor Heitor Vieira Dourado, Manaus, Brazil.

^5^Programa de Pós-Graduação em Medicina Tropical, Universidade do Estado do Amazonas, Manaus, Brazil.

^6^Instituto Nacional de Pesquisa da Amazônia, Manaus, Brazil

^7^Faculdade de Ciência Farmacêuticas, Universidade Federal do Amazonas, Manaus, Brazil

^8^Genomic Health Surveillance Network: Optimization of Assistance and Research in The State of Amazonas – REGESAM, Manaus, Amazonas, Brazil.

**Corresponding author:** Rajendranath Ramasawmy

Faculdade de Medicina - Universidade Nilton Lins

Manaus/Amazonas. Brazil.

Email: [ramasawm@gmail.com](mailto:ramasawm@gmail.com)

| **Supplementary Table 1.** PCR conditions and primer design. | | | | | |
| --- | --- | --- | --- | --- | --- |
| Gene | **SNVs** | **primers** | **MgCl_2_ (mM)** | **Cycling** | **pb** |
| *IL13* | rs1881457 | F: 5’-GCTCCGGGAGTTGCACAGACC-3’  R: 5’-CCAGGCTTGCCCACCCTCTCTTCC-3’ | 2.0 | 95°C 5 min, 40x (95° 15 seg, 68°C 15 seg, 72°C 30 seg) e 72°C 7 min | 275 |
| *IL13* | rs1295687 | F:5’-CCAGCTCCTACTCAGCCATTCC-3’  R:5’-CAGAATGGGTTCATCGTACCTGCC-3’ | 2.0 | 95°C 5 min, 40x (95° 15 seg, 61°C 15 seg, 72°C 30 seg) e 72°C 7 min | 293 |
| *IL13* | rs2069744 | F:5’-GCCAGGCCTGGCCAACACCAGAGAGT-3’  R:5’-GTGGCCTGGGCCACTGTTGAC-3’ | 1.0 | 95°C 5 min, 45x (95° 15 seg, 62°C 15 seg, 72°C 30 seg) e 72°C 7 min | 137 |
| *IL13* | rs2069747 | F:5’-CCCTCTCACACCCACCCTGCACC-3’  R:5’-ACAGATGTGGAAATTGAGGCC-3’ | 1.0 | 95°C 5 min, 45x (95° 15 seg, 61°C 15 seg, 72°C 30 seg) e 72°C 7 min | 191 |
| *IL13* | rs20541 | F:5’-GGCTGAGGTCTAAGCTAAGGAA-3’  R: 5’-ACCAAAATCGAGGTGGCCCAG-3’ | 1.5 | 95°C 5 min, 35x (95° 15 seg, 58°C 15 seg, 72°C 30 seg) e 72°C 5 min | 129 |
| *IL13* | rs1295685  rs848  rs2069750  rs847 | F:5’-GGCAACTGAGGCAGACAGCAG-3’  R:5’-CAGGCCTATCTGTCACAAACT-3’ | 1.5 | 95°C 5 min, 45x (95° 15 seg, 61°C 15 seg, 72°C 30 seg) e 72°C 7 min | 459 |

| **Supplementary Table 2.**  The genotype frequencies and statistical comparisons, based on different genetics models, between patients with *Lg-*CL and healthy controls are provided in. | | | | | | | | |
| --- | --- | --- | --- | --- | --- | --- | --- | --- |
| **Markers** | **Patients with CL (N)** | **%** | **Healthy subjects (N)** | **%** | **OR.adj** | **[CI]** | **Adj.*p*-value** | **AIC** |
| rs1881457 |  |  |  |  |  |  |  |  |
| Codominant |  |  |  |  |  |  |  |  |
| A/A | 529 | 61.9 | 540 | 67.9 | 1.00 |  | 0.3012 | 2311 |
| A/C | 273 | 31.9 | 279 | 32.5 | 1.05 | [0.85 - 1.29] |  |  |
| C/C | 53 | 6.2 | 40 | 4.7 | 0.73 | [0.47 - 1.14] |  |  |
| Dominant |  |  |  |  |  |  |  |  |
| A/A | 529 | 61.9 | 540 | 62.9 | 1.00 |  | 0.9653 | 2312 |
| A/C-C/C | 326 | 38.1 | 319 | 37.1 | 1.00 | [0.81 - 1.22] |  |  |
| Recessive |  |  |  |  |  |  |  |  |
| A/A-A/C | 802 | 93.8 | 819 | 95.3 | 1.00 |  | 0.1369 | 2309 |
| C/C | 53 | 6.2 | 40 | 4.7 | 0.72 | [0.47 - 1.11] |  |  |
| Overdominant |  |  |  |  |  |  |  |  |
| A/A-C/C | 582 | 68.1 | 580 | 67.5 | 1.00 |  | 0.5000 | 2311 |
| A/C | 273 | 31.9 | 279 | 32.5 | 1.07 | [0.87 - 1.32] |  |  |
| rs1295687 |  |  |  |  |  |  |  |  |
| Codominant |  |  |  |  |  |  |  |  |
| C/C | 593 | 69.4 | 587 | 68.3 | 1.00 |  | 0.7015 | 2313 |
| C/G | 228 | 26.7 | 239 | 27.8 | 1.08 | [0.87 - 1.35] |  |  |
| G/G | 34 | 4.0 | 33 | 3.8 | 0.91 | [0.55 - 1.51] |  |  |
| Dominant |  |  |  |  |  |  |  |  |
| C/C | 593 | 69.4 | 587 | 68.3 | 1.00 |  | 0.5835 | 2311 |
| C/G-G/G | 262 | 30.6 | 272 | 31.7 | 1.06 | [0.86 - 1.31] |  |  |
| Recessive |  |  |  |  |  |  |  |  |
| C/C-C/G | 821 | 96.0 | 826 | 96.2 | 1.00 |  | 0.6566 | 2311 |
| G/G | 34 | 4.0 | 33 | 3.8 | 0.89 | [0.54 - 1.47] |  |  |
| Overdominant |  |  |  |  |  |  |  |  |
| C/C-G/G | 627 | 73.3 | 620 | 72.2 | 1.00 |  | 0.4447 | 2311 |
| G/C | 228 | 26.7 | 239 | 27.8 | 1.09 | [0.88 - 1.35] |  |  |
| rs2069744 |  |  |  |  |  |  |  |  |
| Codominant |  |  |  |  |  |  |  |  |
| C/C | 679 | 79.4 | 663 | 77.2 | 1.00 |  | 0.3279 | 2311 |
| T/C | 162 | 18.9 | 178 | 20.7 | 1.13 | [0.89 - 1.44] |  |  |
| T/T | 14 | 1.6 | 18 | 2.1 | 1.54 | [0.75 - 3.14] |  |  |
| Dominant |  |  |  |  |  |  |  |  |
| C/C | 679 | 79.4 | 663 | 77.2 | 1.00 |  | 0.2098 | 2310 |
| T/C-T/T | 176 | 20.6 | 196 | 22.8 | 1.16 | [0.92 - 1.47] |  |  |
| Recessive |  |  |  |  |  |  |  |  |
| C/C-T/C | 841 | 98.4 | 841 | 97.9 | 1.00 |  | 0.2659 | 2310 |
| T/T | 14 | 1.6 | 18 | 2.1 | 1.50 | [0.73 - 3.06] |  |  |
| Overdominant |  |  |  |  |  |  |  |  |
| C/C-T/T | 693 | 81.1 | 681 | 79.3 | 1.00 |  | 0.3596 | 2311 |
| T/C | 162 | 18.9 | 178 | 20.7 | 1.12 | [0.88 - 1.43] |  |  |
| rs2069747 |  |  |  |  |  |  |  |  |
| Codominant |  |  |  |  |  |  |  |  |
| C/C | 829 | 97.0 | 837 | 97.4 | 1.00 |  | 0.5512 | 2311 |
| C/T | 26 | 3.0 | 22 | 2.6 | 0.84 | [0.46 - 1.51] |  |  |
| rs20541 |  |  |  |  |  |  |  |  |
| Codominant |  |  |  |  |  |  |  |  |
| G/G | 358 | 41.9 | 341 | 39.7 | 1.00 |  | 0.3940 | 2312 |
| A/G | 374 | 43.7 | 382 | 44.5 | 1.09 | [0.88 - 1.34] |  |  |
| A/A | 123 | 14.4 | 136 | 15.8 | 1.22 | [0.91 - 1.63] |  |  |
| Dominant |  |  |  |  |  |  |  |  |
| G/G | 358 | 38.5 | 341 | 39.7 | 1.00 |  | 0.2647 | 2310 |
| A/G-A/A | 497 | 58.1 | 518 | 60.3 | 1.12 | [0.92 - 1.36] |  |  |
| Recessive |  |  |  |  |  |  |  |  |
| G/G-A/G | 732 | 85.6 | 723 | 83.4 | 1.00 |  | 0.2596 | 2310 |
| A/A | 123 | 14.4 | 136 | 15.8 | 1.17 | [0.89 - 1.53] |  |  |
| Overdominant |  |  |  |  |  |  |  |  |
| G/G-A/A | 481 | 56.3 | 477 | 55.5 | 1.00 |  | 0.7723 | 2311 |
| A/G | 374 | 43.7 | 382 | 44.5 | 1.03 | [0.85 - 1.25] |  |  |
| rs1295685 |  |  |  |  |  |  |  |  |
| Codominant |  |  |  |  |  |  |  |  |
| G/G | 346 | 40.5 | 337 | 39.2 | 1.00 |  | 0.7496 | 2313 |
| A/G | 387 | 45.3 | 396 | 46.1 | 1.08 | [0.87 - 1.33] |  |  |
| A/A | 122 | 14.3 | 126 | 14.7 | 1.09 | [0.81 - 1.46] |  |  |
| Dominant |  |  |  |  |  |  |  |  |
| G/G | 346 | 40.5 | 337 | 39.2 | 1.00 |  | 0.4500 | 2311 |
| A/G-A/A | 509 | 59.5 | 522 | 60.8 | 1.08 | [0.89 - 1.32] |  |  |
| Recessive |  |  |  |  |  |  |  |  |
| G/G-A/G | 733 | 85.7 | 733 | 85.3 | 1.00 |  | 0.7447 | 2311 |
| A/A | 122 | 14.3 | 126 | 14.7 | 1.05 | [0.80 - 1.38] |  |  |
| Overdominant |  |  |  |  |  |  |  |  |
| G/G-A/A | 468 | 54.7 | 463 | 53.9 | 1.00 |  | 0.6086 | 2311 |
| A/G | 387 | 45.3 | 936 | 46.1 | 1.05 | [0.87 - 1.28] |  |  |
| rs848 |  |  |  |  |  |  |  |  |
| Codominant |  |  |  |  |  |  |  |  |
| C/C | 293 | 34.3 | 268 | 31.2 | 1.00 |  | 0.2996 | 2311 |
| A/C | 404 | 47.3 | 416 | 48.4 | 1.14 | [0.91 - 1.42] |  |  |
| A/A | 158 | 18.5 | 175 | 20.4 | 1.23 | [0.93 - 1.62] |  |  |
| Dominant |  |  |  |  |  |  |  |  |
| C/C | 293 | 34.3 | 268 | 31.2 | 1.00 |  | 0.1513 | 2310 |
| A/C-A/A | 562 | 65.7 | 591 | 68.8 | 1.16 | [0.95 - 1.43] |  |  |
| Recessive |  |  |  |  |  |  |  |  |
| C/C-A/C | 697 | 81.5 | 684 | 79.6 | 1.00 |  | 0.2942 | 2310 |
| A/A | 158 | 18.5 | 175 | 20.4 | 1.14 | [0.89 - 1.46] |  |  |
| Overdominant |  |  |  |  |  |  |  |  |
| C/C-A/A | 451 | 52.7 | 443 | 51.6 | 1.00 |  | 0.6052 | 2311 |
| A/C | 404 | 47.3 | 416 | 48.4 | 1.05 | [0.87 - 1.28] |  |  |
| rs2069750 |  |  |  |  |  |  |  |  |
| Codominant |  |  |  |  |  |  |  |  |
| G/G | 825 | 96.5 | 813 | 94.6 | 1.00 |  | 0.3147 | 2311 |
| G/C | 29 | 3.4 | 45 | 5.2 | 1.45 | [0.89 - 2.37] |  |  |
| C/C | 1 | 0.1 | 1 | 0.1 | 1.13 | [0.07 - 18.3] |  |  |
| Dominant |  |  |  |  |  |  |  |  |
| G/G | 825 | 96.5 | 813 | 94.6 | 1.00 |  | 0.1308 | 2309 |
| G/C-C/C | 30 | 3.5 | 46 | 5.4 | 1.44 | [0.89 - 2.33] |  |  |
| Recessive |  |  |  |  |  |  |  |  |
| G/G-G/C | 854 | 99.9 | 858 | 99.9 | 1.00 |  | 0.9382 | 2312 |
| C/C | 1 | 0.1 | 1 | 0.1 | 1.12 | [0.07 - 18.0] |  |  |
| Overdominant |  |  |  |  |  |  |  |  |
| G/G-C/C | 826 | 96.6 | 814 | 94.8 | 1.00 |  | 0.1290 | 2309 |
| G/C | 29 | 3.4 | 45 | 5.2 | 1.45 | [0.89 - 2.37] |  |  |
| rs847 |  |  |  |  |  |  |  |  |
| Codominant |  |  |  |  |  |  |  |  |
| C/C | 336 | 39.3 | 331 | 38.5 | 1.00 |  | 0.8594 | 2313 |
| T/C | 388 | 45.4 | 394 | 45.9 | 1.05 | [0.85 - 1.30] |  |  |
| T/T | 131 | 15.3 | 184 | 15.9 | 1.06 | [0.80 - 1.42] |  |  |
| Dominant |  |  |  |  |  |  |  |  |
| C/C | 336 | 39.3 | 331 | 38.5 | 1.00 |  | 0.5841 | 2311 |
| T/C-T/T | 519 | 60.7 | 528 | 61.5 | 1.06 | [0.87 - 1.29] |  |  |
| Recessive |  |  |  |  |  |  |  |  |
| C/C-T/C | 724 | 84.7 | 725 | 84.4 | 1.00 |  | 0.8094 | 2312 |
| T/T | 131 | 15.3 | 134 | 15.6 | 1.03 | [0.79 - 1.35] |  |  |
| Overdominant |  |  |  |  |  |  |  |  |
| C/C-T/T | 467 | 54.6 | 465 | 54.1 | 1.00 |  | 0.7185 | 2311 |
| T/C | 388 | 45.4 | 394 | 45.9 | 1.04 | [0.85 - 1.26] |  |  |

| **Supplementary Table 3**. The distribution of the haplotypes frequencies as derived from the nine single nucleotide variants of the *IL13* gene among the patients with *Lg*-CL (cases) and healthy controls (HC). | | | | | | | |
| --- | --- | --- | --- | --- | --- | --- | --- |
| **Hap** | **Haplotype** | **Cases**  **(%)** | **HC**  **(%)** | ***p*.value [OR (95%CI)]** | | ***Adj* *p*-value [OR (95% CI)]** |  |
| 1 | ACCCCGCGC | 648 (38) | 612 (37) | 0.21 [1.09 (0.9-1.2)] | 0.13 [1.00 (0.00-0.00)] | |  |
| 2 | AGCCTAAGT | 127 (7.6) | 130 (7.8) | 0.85 [0.98 (0.7-1.2)] | 0.65 [0.78 (0.58-1.05)] | |  |
| 3 | CCCCTAAGT | 131 (7.9) | 118 (7.1) | 0.39 [1.10 (0.8-1.4)] | 0.48 [1.10 (0.81-1.52)] | |  |
| 4 | ACCCTAAGT | 133 (8) | 115 (7) | 0.22 [1.20 (0.9-1.5)] | 0.19 [1.08 (0.79-1.47)] | |  |
| 5 | CCCCCGCGC | 75 (4.5) | 65 (4) | 0.33 [1.10 (0.8-1.6)] | 0.29 [1.21 (0.76-1.92)] | |  |
| 6 | ACTCCGCGC | 61 (3.7) | 57 (3.4) | 0.70 [1.10 (0.7-1.5)] | 0.95 [1.14 (0.72-1.75)] | |  |
| 7 | AGCCCGCGC | 63 (3.8) | 43 (2.6) | 0.04 [1.40 (0.9-2.2)] | 0.01 [1.69 (1.03-2.78)] | |  |
| 8 | ACCCTGCGC | 77 (4.6) | 29 (1.8) | 3.0e-6 [2.8 (1.7-4.2)] | 1.6e-6 [2.85 (1.67-4.89)] | |  |
| 9 | CCTCTAAGT | 48 (3) | 51 (3.1) | 0.74 [0.90 (0.6-1.3)] | 0.76 [0.81 (0.50-1.31)] | |  |
| 10 | ACCCCGCGT | 6 (0.4) | 88 (5.3) | 1.3e-17 [0.06 (0.03-0.14)] | 6.1e-19 [0.02 (0.00-0.07)] | |  |
| 11 | ACCCCAAGT | 58 (3.4) | 23 (1.4) | 0.72e-5 [2.5 (1.5-4.1)] | 3.2e-5 [2.68 (1.47-4.79)] | |  |
| 12 | AGCCTAAGC | 2 (0.1) | 46 (2.8) | 1.1e-10 [0.04 (0.01-0.15)] | 7.8e-12 [0.00 (0.00-0.00)] | |  |
| 13 | CCCCTAAGC | 3 (0.2) | 38 (2.3) | 2.5e-8 [0.07 (0.02-0.2)] | 9.5e-11 [0.00 (0.00-0.00)] | |  |
| 14 | ACCCCGAGC | 16 (1) | 21 (1.2) | 0.45 [0.80 (0.4-1.4)] | 0.50 [0.67 (0.30-1.50)] | |  |
| Hap: haplotypes; HC: healthy controls; OR: odds ratio, CI 95%: confidence interval, adj.: sex and age ajusted. p-value < 0.05 are considered significant. | | | | | | |  |

| **Supplementary Table 4.** Correlations of Interleukin-4 circulating plasma concentrations with *IL13* SNVs in Patients with CL, Healthy Subjects, and Totals. | | | | | | | | | | | | | | | |
| --- | --- | --- | --- | --- | --- | --- | --- | --- | --- | --- | --- | --- | --- | --- | --- |
|  | Patients with CL | | | | | Healthy Subjects | | | | | Totals | | | | |
| rs1881457 | n | me | se | p-value | AIC | n | me | se | p-value | AIC | n | me | se | p-value | AIC |
| Codominant |  |  |  |  |  |  |  |  |  |  |  |  |  |  |  |
| A/A | 193 | -0.8988 | 0.05383 | 0.3781 | 708.0 | 229 | -1.374 | 0.03281 | 0.7319 | 546.8 | 422 | -1.157 | 0.03246 | 0.6665 | 1348 |
| A/C | 111 | -1.0032 | 0.06639 |  |  | 112 | -1.375 | 0.05394 |  |  | 223 | -1.190 | 0.04442 |  |  |
| C/C | 15 | -1.0254 | 0.16363 |  |  | 14 | -1.266 | 0.10192 |  |  | 29 | -1.141 | 0.09883 |  |  |
| Dominant |  |  |  |  |  |  |  |  |  |  |  |  |  |  |  |
| A/A | 193 | -0.8988 | 0.05383 | 0.1628 | 706.0 | 229 | -1.374 | 0.03281 | 0.8577 | 545.4 | 422 | -1.157 | 0.03246 | 0.4558 | 1347 |
| A/C-C/C | 126 | -1.0058 | 0.06143 |  |  | 126 | -1.363 | 0.04925 |  |  | 252 | -1.184 | 0.04088 |  |  |
| Recessive |  |  |  |  |  |  |  |  |  |  |  |  |  |  |  |
| A/A-A/C | 304 | -0.9369 | 0.04193 | 0.7151 | 707.8 | 341 | -1.374 | 0.02823 | 0.4297 | 544.8 | 645 | -1.168 | 0.02620 | 0.7786 | 1347 |
| C/C | 15 | -1.0254 | 0.16363 |  |  | 14 | -1.266 | 0.10192 |  |  | 29 | -1.141 | 0.09883 |  |  |
| Overdominant |  |  |  |  |  |  |  |  |  |  |  |  |  |  |  |
| A/A-C/C | 208 | -0.9079 | 0.05128 | 0.2046 | 706.3 | 243 | -1.368 | 0.03147 | 0.8840 | 545.4 | 451 | -1.156 | 0.03101 | 0.3743 | 1346 |
| A/C | 111 | -1.0032 | 0.06639 |  |  | 112 | -1.375 | 0.05394 |  |  | 223 | -1.190 | 0.04442 |  |  |
| rs1295687 |  |  |  |  |  |  |  |  |  |  |  |  |  |  |  |
| Codominant |  |  |  |  |  |  |  |  |  |  |  |  |  |  |  |
| C/C | 217 | -0.9312 | 0.05040 | 0.2713 | 707.3 | 237 | -1.356 | 0.03255 | 0.6293 | 546.5 | 454 | -1.153 | 0.03109 | 0.6081 | 1348 |
| C/G | 87 | -0.9129 | 0.07010 |  |  | 103 | -1.409 | 0.05451 |  |  | 190 | -1.182 | 0.04708 |  |  |
| G/G | 15 | -1.2479 | 0.22187 |  |  | 15 | -1.331 | 0.13301 |  |  | 30 | -1.289 | 0.12732 |  |  |
| Dominant |  |  |  |  |  |  |  |  |  |  |  |  |  |  |  |
| C/C | 217 | -0.9312 | 0.05040 | 0.7153 | 707.8 | 237 | -1.356 | 0.03255 | 0.4585 | 544.9 | 454 | -1.153 | 0.03109 | 0.4484 | 1347 |
| C/G-G/G | 102 | -0.9622 | 0.06864 |  |  | 118 | -1.399 | 0.05036 |  |  | 220 | -1.197 | 0.04417 |  |  |
| Recessive |  |  |  |  |  |  |  |  |  |  |  |  |  |  |  |
| C/C-C/G | 304 | -0.9259 | 0.04113 | 0.1078 | 705.3 | 340 | -1.372 | 0.02805 | 0.7138 | 545.3 | 644 | -1.161 | 0.02593 | 0.3945 | 1346 |
| G/G | 15 | -1.2479 | 0.22187 |  |  | 15 | -1.331 | 0.13301 |  |  | 30 | -1.289 | 0.12732 |  |  |
| Overdominant |  |  |  |  |  |  |  |  |  |  |  |  |  |  |  |
| C/C-G/G | 232 | -0.9516 | 0.04941 | 0.7040 | 707.8 | 252 | -1.354 | 0.03156 | 0.3515 | 544.6 | 484 | -1.161 | 0.03021 | 0.6890 | 1347 |
| C/G | 87 | -0.9129 | 0.07010 |  |  | 103 | -1.409 | 0.05451 |  |  | 190 | -1.182 | 0.04708 |  |  |
| rs2069744 |  |  |  |  |  |  |  |  |  |  |  |  |  |  |  |
| Codominant |  |  |  |  |  |  |  |  |  |  |  |  |  |  |  |
| C/C | 261 | -0.8974 | 0.04392 | 0.05321 | 704.0 | 278 | -1.384 | 0.03031 | 0.3845 | 545.5 | 539 | -1.148 | 0.02838 | 0.24572 | 1346 |
| C/T | 55 | -1.1177 | 0.10496 |  |  | 70 | -1.341 | 0.06736 |  |  | 125 | -1.243 | 0.06020 |  |  |
| T/T | 3 | -1.5006 | 0.38850 |  |  | 7 | -1.103 | 0.16676 |  |  | 10 | -1.223 | 0.16346 |  |  |
| Dominant |  |  |  |  |  |  |  |  |  |  |  |  |  |  |  |
| C/C | 261 | -0.8974 | 0.04392 | 0.02671 | 703.0 | 278 | -1.384 | 0.03031 | 0.4150 | 544.8 | 539 | -1.148 | 0.02838 | 0.09397 | 1344 |
| C/T-T/T | 58 | -1.1375 | 0.10148 |  |  | 77 | -1.320 | 0.06329 |  |  | 135 | -1.241 | 0.05691 |  |  |
| Recessive |  |  |  |  |  |  |  |  |  |  |  |  |  |  |  |
| C/C-C/T | 316 | -0.9358 | 0.04082 | 0.15789 | 705.9 | 348 | -1.375 | 0.02771 | 0.1960 | 543.7 | 664 | -1.166 | 0.02569 | 0.63923 | 1347 |
| T/T | 3 | -1.5006 | 0.38850 |  |  | 7 | -1.103 | 0.16676 |  |  | 10 | -1.223 | 0.16346 |  |  |
| Overdominant |  |  |  |  |  |  |  |  |  |  |  |  |  |  |  |
| C/C-T/T | 264 | -0.9043 | 0.04375 | 0.05762 | 704.3 | 285 | -1.377 | 0.02992 | 0.6971 | 545.3 | 549 | -1.150 | 0.02801 | 0.11478 | 1345 |
| C/T | 55 | -1.1177 | 0.10496 |  |  | 70 | -1.341 | 0.06736 |  |  | 125 | -1.243 | 0.06020 |  |  |
| rs2069747 |  |  |  |  |  |  |  |  |  |  |  |  |  |  |  |
| Codominant |  |  |  |  |  |  |  |  |  |  |  |  |  |  |  |
| C/C | 307 | -0.9289 | 0.04099 | 0.1335 | 705.7 | 348 | -1.374 | 0.0277 | 0.2722 | 544.2 | 655 | -1.166 | 0.0257 | 0.6856 | 1347 |
| C/T | 12 | -1.2533 | 0.25568 |  |  | 7 | -1.156 | 0.1836 |  |  | 19 | -1.217 | 0.1717 |  |  |
| rs20541 |  |  |  |  |  |  |  |  |  |  |  |  |  |  |  |
| Codominant |  |  |  |  |  |  |  |  |  |  |  |  |  |  |  |
| G/G | 119 | -0.8870 | 0.06550 | 0.12134 | 705.7 | 154 | -1.333 | 0.04625 | 0.4937 | 546.0 | 273 | -1.138 | 0.04086 | 0.19878 | 1346 |
| A/G | 150 | -1.0299 | 0.06174 |  |  | 154 | -1.406 | 0.03821 |  |  | 304 | -1.220 | 0.03762 |  |  |
| A/A | 50 | -0.8033 | 0.08983 |  |  | 47 | -1.375 | 0.06525 |  |  | 97 | -1.080 | 0.06295 |  |  |
| Dominant |  |  |  |  |  |  |  |  |  |  |  |  |  |  |  |
| G/G | 119 | -0.8870 | 0.06550 | 0.31738 | 707.0 | 154 | -1.333 | 0.04625 | 0.2507 | 544.1 | 273 | -1.138 | 0.04086 | 0.39656 | 1346 |
| A/G-A/A | 200 | -0.9732 | 0.05182 |  |  | 201 | -1.399 | 0.03295 |  |  | 401 | -1.187 | 0.03243 |  |  |
| Recessive |  |  |  |  |  |  |  |  |  |  |  |  |  |  |  |
| A/G-G/G | 269 | -0.9667 | 0.04512 | 0.17564 | 706.1 | 308 | -1.369 | 0.03002 | 0.9161 | 545.4 | 577 | -1.182 | 0.02771 | 0.22924 | 1346 |
| A/A | 50 | -0.8033 | 0.08983 |  |  | 47 | -1.375 | 0.06525 |  |  | 97 | -1.080 | 0.06295 |  |  |
| Overdominant |  |  |  |  |  |  |  |  |  |  |  |  |  |  |  |
| G/G-A/A | 169 | -0.8623 | 0.05317 | 0.05011 | 704.1 | 201 | -1.343 | 0.03853 | 0.2820 | 544.3 | 370 | -1.123 | 0.03435 | 0.09165 | 1344 |
| A/G | 150 | -1.0299 | 0.06174 |  |  | 154 | -1.406 | 0.03821 |  |  | 304 | -1.220 | 0.03762 |  |  |
| rs1295685 |  |  |  |  |  |  |  |  |  |  |  |  |  |  |  |
| Codominant |  |  |  |  |  |  |  |  |  |  |  |  |  |  |  |
| G/G | 125 | -0.8798 | 0.06823 | 0.23240 | 707.0 | 142 | -1.335 | 0.04599 | 0.5831 | 546.3 | 267 | -1.122 | 0.04250 | 0.2585 | 1346 |
| A/G | 153 | -1.0156 | 0.05694 |  |  | 166 | -1.394 | 0.03760 |  |  | 319 | -1.213 | 0.03518 |  |  |
| A/A | 41 | -0.8500 | 0.10608 |  |  | 47 | -1.391 | 0.07775 |  |  | 88 | -1.139 | 0.07039 |  |  |
| Dominant |  |  |  |  |  |  |  |  |  |  |  |  |  |  |  |
| G/G | 125 | -0.8798 | 0.06823 | 0.24102 | 706.6 | 142 | -1.335 | 0.04599 | 0.2987 | 544.3 | 267 | -1.122 | 0.04250 | 0.1594 | 1345 |
| A/G-A/A | 194 | -0.9806 | 0.05031 |  |  | 213 | -1.394 | 0.03386 |  |  | 407 | -1.197 | 0.03149 |  |  |
| Recessive |  |  |  |  |  |  |  |  |  |  |  |  |  |  |  |
| G/G-A/G | 278 | -0.9545 | 0.04396 | 0.41340 | 707.3 | 308 | -1.367 | 0.02933 | 0.7499 | 545.3 | 586 | -1.171 | 0.02727 | 0.7138 | 1347 |
| A/A | 41 | -0.8500 | 0.10608 |  |  | 47 | -1.391 | 0.07775 |  |  | 88 | -1.139 | 0.07039 |  |  |
| Overdominant |  |  |  |  |  |  |  |  |  |  |  |  |  |  |  |
| G/G-A/A | 166 | -0.8724 | 0.05752 | 0.08972 | 705.0 | 189 | -1.349 | 0.03953 | 0.4217 | 544.8 | 355 | -1.126 | 0.03637 | 0.1038 | 1344 |
| A/G | 153 | -1.0156 | 0.05694 |  |  | 166 | -1.394 | 0.03760 |  |  | 319 | -1.213 | 0.03518 |  |  |
| rs848 |  |  |  |  |  |  |  |  |  |  |  |  |  |  |  |
| Codominant |  |  |  |  |  |  |  |  |  |  |  |  |  |  |  |
| C/C | 104 | -0.8312 | 0.07071 | 0.16113 | 706.3 | 113 | -1.315 | 0.04991 | 0.4110 | 545.6 | 217 | -1.083 | 0.04567 | 0.08846 | 1344 |
| A/C | 162 | -1.0071 | 0.05647 |  |  | 175 | -1.397 | 0.03803 |  |  | 337 | -1.210 | 0.03516 |  |  |
| A/A | 53 | -0.9549 | 0.10256 |  |  | 67 | -1.392 | 0.06444 |  |  | 120 | -1.199 | 0.06093 |  |  |
| Dominant |  |  |  |  |  |  |  |  |  |  |  |  |  |  |  |
| C/C | 104 | -0.8312 | 0.07071 | 0.06302 | 704.5 | 113 | -1.315 | 0.04991 | 0.1822 | 543.6 | 217 | -1.083 | 0.04567 | 0.02787 | 1342 |
| A/C-A/A | 215 | -0.9942 | 0.04940 |  |  | 242 | -1.396 | 0.03271 |  |  | 457 | -1.207 | 0.03043 |  |  |
| Recessive |  |  |  |  |  |  |  |  |  |  |  |  |  |  |  |
| C/C-A/C | 266 | -0.9383 | 0.04435 | 0.87773 | 707.9 | 288 | -1.365 | 0.03033 | 0.6846 | 545.3 | 554 | -1.160 | 0.02798 | 0.56365 | 1347 |
| A/A | 53 | -0.9549 | 0.10256 |  |  | 67 | -1.392 | 0.06444 |  |  | 120 | -1.199 | 0.06093 |  |  |
| Overdominant |  |  |  |  |  |  |  |  |  |  |  |  |  |  |  |
| C/C-A/A | 157 | -0.8729 | 0.05824 | 0.10366 | 705.3 | 180 | -1.344 | 0.03945 | 0.3559 | 544.6 | 337 | -1.124 | 0.03662 | 0.10741 | 1344 |
| A/C | 162 | -1.0071 | 0.05647 |  |  | 175 | -1.397 | 0.03803 |  |  | 337 | -1.210 | 0.03516 |  |  |
| rs2069750 |  |  |  |  |  |  |  |  |  |  |  |  |  |  |  |
| Codominant |  |  |  |  |  |  |  |  |  |  |  |  |  |  |  |
| G/G | 305 | -0.9337 | 0.04146 | 0.4512 | 707.4 | 332 | -1.361 | 0.02866 | 0.1925 | 543.7 | 637 | -1.156 | 0.02623 | 0.1072 | 1345 |
| C/G | 14 | -1.1024 | 0.20830 |  |  | 23 | -1.500 | 0.08618 |  |  | 37 | -1.349 | 0.09890 |  |  |
| rs847 |  |  |  |  |  |  |  |  |  |  |  |  |  |  |  |
| Codominant |  |  |  |  |  |  |  |  |  |  |  |  |  |  |  |
| C/C | 125 | -0.8543 | 0.06729 | 0.14615 | 706.1 | 129 | -1.339 | 0.04343 | 0.4509 | 545.8 | 254 | -1.100 | 0.04253 | 0.025601 | 1342 |
| C/T | 150 | -1.0280 | 0.05808 |  |  | 185 | -1.401 | 0.03841 |  |  | 335 | -1.234 | 0.03501 |  |  |
| T/T | 44 | -0.8912 | 0.10259 |  |  | 41 | -1.328 | 0.08795 |  |  | 85 | -1.102 | 0.07165 |  |  |
| Dominant |  |  |  |  |  |  |  |  |  |  |  |  |  |  |  |
| C/C | 125 | -0.8543 | 0.06729 | 0.09691 | 705.2 | 129 | -1.339 | 0.04343 | 0.3683 | 544.6 | 254 | -1.100 | 0.04253 | 0.034218 | 1343 |
| C/T-T/T | 194 | -0.9970 | 0.05062 |  |  | 226 | -1.388 | 0.03522 |  |  | 420 | -1.207 | 0.03153 |  |  |
| Recessive |  |  |  |  |  |  |  |  |  |  |  |  |  |  |  |
| C/C-C/T | 275 | -0.9490 | 0.04426 | 0.65076 | 707.8 | 314 | -1.376 | 0.02882 | 0.5471 | 545.1 | 589 | -1.176 | 0.02719 | 0.326087 | 1346 |
| T/T | 44 | -0.8912 | 0.10259 |  |  | 41 | -1.328 | 0.08795 |  |  | 85 | -1.102 | 0.07165 |  |  |
| Overdominant |  |  |  |  |  |  |  |  |  |  |  |  |  |  |  |
| C/C-T/T | 169 | -0.8639 | 0.05635 | 0.05242 | 704.2 | 170 | -1.336 | 0.03906 | 0.2094 | 543.8 | 339 | -1.101 | 0.03653 | 0.006766 | 1340 |
| C/T | 150 | -1.0280 | 0.05808 |  |  | 185 | -1.401 | 0.03841 |  |  | 335 | -1.234 | 0.03501 |  |  |
| **me:** mean in picogram per milliliters; **se:** standard error of mean; **p-value:** sex and age adjusted; **AIC:** Akaike Information Criterium for each inheritance models. p-value < 0.05 are considered significant. | | | | | | | | | | | | | | | |

| **Supplementary Table 5.** Correlations of Interleukin-5 circulating plasma concentrations with *IL13* SNVs in Patients with CL, Healthy Subjects, and Totals. | | | | | | | | | | | | | | | |
| --- | --- | --- | --- | --- | --- | --- | --- | --- | --- | --- | --- | --- | --- | --- | --- |
|  | Patients with CL | | | | | Healthy Subjects | | | | | Totals | | | | |
| rs1881457 | n | me | se | p-value | AIC | n | me | se | p-value | AIC | n | me | se | p-value | AIC |
| Codominant |  |  |  |  |  |  |  |  |  |  |  |  |  |  |  |
| A/A | 135 | 1.452 | 0.09087 | 0.6174 | 649.4 | 197 | 1.0407 | 0.04772 | 0.14269 | 660.0 | 332 | 1.208 | 0.04778 | 0.2598 | 1358 |
| A/C | 83 | 1.448 | 0.10276 |  |  | 90 | 0.8881 | 0.08815 |  |  | 173 | 1.157 | 0.07044 |  |  |
| C/C | 9 | 1.779 | 0.25480 |  |  | 11 | 1.2298 | 0.17496 |  |  | 20 | 1.477 | 0.15842 |  |  |
| Dominant |  |  |  |  |  |  |  |  |  |  |  |  |  |  |  |
| A/A | 135 | 1.452 | 0.09087 | 0.9849 | 648.3 | 197 | 1.0407 | 0.04772 | 0.19039 | 660.2 | 332 | 1.208 | 0.04778 | 0.6546 | 1359 |
| A/C-C/C | 92 | 1.480 | 0.09618 |  |  | 101 | 0.9253 | 0.08130 |  |  | 193 | 1.190 | 0.06551 |  |  |
| Recessive |  |  |  |  |  |  |  |  |  |  |  |  |  |  |  |
| A/A-A/C | 218 | 1.450 | 0.06839 | 0.3378 | 647.4 | 287 | 0.9929 | 0.04298 | 0.28985 | 660.8 | 505 | 1.190 | 0.03958 | 0.1591 | 1357 |
| C/C | 9 | 1.779 | 0.25480 |  |  | 11 | 1.2298 | 0.17496 |  |  | 20 | 1.477 | 0.15842 |  |  |
| Overdominant |  |  |  |  |  |  |  |  |  |  |  |  |  |  |  |
| A/A-C/C | 144 | 1.472 | 0.08674 | 0.7126 | 648.2 | 208 | 1.0507 | 0.04614 | 0.07382 | 658.7 | 352 | 1.223 | 0.04603 | 0.3016 | 1358 |
| A/C | 83 | 1.448 | 0.10276 |  |  | 90 | 0.8881 | 0.08815 |  |  | 173 | 1.157 | 0.07044 |  |  |
| rs1295687 |  |  |  |  |  |  |  |  |  |  |  |  |  |  |  |
| Codominant |  |  |  |  |  |  |  |  |  |  |  |  |  |  |  |
| C/C | 160 | 1.447 | 0.07941 | 0.4295 | 648.6 | 198 | 1.0147 | 0.05054 | 0.5280 | 662.6 | 358 | 1.208 | 0.04652 | 0.9726 | 1361 |
| C/G | 55 | 1.577 | 0.13623 |  |  | 87 | 0.9470 | 0.08223 |  |  | 142 | 1.191 | 0.07712 |  |  |
| G/G | 12 | 1.160 | 0.26745 |  |  | 13 | 1.1685 | 0.17350 |  |  | 25 | 1.164 | 0.15350 |  |  |
| Dominant |  |  |  |  |  |  |  |  |  |  |  |  |  |  |  |
| C/C | 160 | 1.447 | 0.07941 | 0.6991 | 648.2 | 198 | 1.0147 | 0.05054 | 0.6623 | 661.7 | 358 | 1.208 | 0.04652 | 0.8149 | 1359 |
| C/G-G/G | 67 | 1.502 | 0.12242 |  |  | 100 | 0.9758 | 0.07511 |  |  | 167 | 1.187 | 0.06932 |  |  |
| Recessive |  |  |  |  |  |  |  |  |  |  |  |  |  |  |  |
| C/C-C/G | 215 | 1.480 | 0.06856 | 0.3102 | 647.3 | 285 | 0.9940 | 0.04312 | 0.3892 | 661.2 | 500 | 1.203 | 0.03983 | 0.9181 | 1359 |
| G/G | 12 | 1.160 | 0.26745 |  |  | 13 | 1.1685 | 0.17350 |  |  | 25 | 1.164 | 0.15350 |  |  |
| Overdominant |  |  |  |  |  |  |  |  |  |  |  |  |  |  |  |
| C/C-G/G | 172 | 1.427 | 0.07620 | 0.3465 | 647.4 | 211 | 1.0241 | 0.04859 | 0.3998 | 661.2 | 383 | 1.205 | 0.04458 | 0.8445 | 1359 |
| C/G | 55 | 1.577 | 0.13623 |  |  | 87 | 0.9470 | 0.08223 |  |  | 142 | 1.191 | 0.07712 |  |  |
| rs2069744 |  |  |  |  |  |  |  |  |  |  |  |  |  |  |  |
| Codominant |  |  |  |  |  |  |  |  |  |  |  |  |  |  |  |
| C/C | 188 | 1.538 | 0.07053 | 0.034949 | 643.5 | 232 | 1.0092 | 0.04802 | 0.2728 | 661.3 | 420 | 1.246 | 0.04314 | 0.024318 | 1354 |
| C/T | 38 | 1.099 | 0.18057 |  |  | 59 | 0.9262 | 0.09369 |  |  | 97 | 0.994 | 0.09068 |  |  |
| T/T | 1 | 1.221 | 0.00000 |  |  | 7 | 1.3855 | 0.11707 |  |  | 8 | 1.365 | 0.10346 |  |  |
| Dominant |  |  |  |  |  |  |  |  |  |  |  |  |  |  |  |
| C/C | 188 | 1.538 | 0.07053 | 0.009612 | 641.5 | 232 | 1.0092 | 0.04802 | 0.7090 | 661.8 | 420 | 1.246 | 0.04314 | 0.011239 | 1353 |
| C/T-T/T | 39 | 1.102 | 0.17591 |  |  | 66 | 0.9749 | 0.08628 |  |  | 105 | 1.022 | 0.08462 |  |  |
| Recessive |  |  |  |  |  |  |  |  |  |  |  |  |  |  |  |
| C/C-C/T | 226 | 1.464 | 0.06678 | 0.645635 | 648.1 | 291 | 0.9924 | 0.04271 | 0.1628 | 659.9 | 517 | 1.199 | 0.03916 | 0.727646 | 1359 |
| T/T | 1 | 1.221 | 0.00000 |  |  | 7 | 1.3855 | 0.11707 |  |  | 8 | 1.365 | 0.10346 |  |  |
| Overdominant |  |  |  |  |  |  |  |  |  |  |  |  |  |  |  |
| C/C-T/T | 189 | 1.537 | 0.07017 | 0.011536 | 641.8 | 239 | 1.0202 | 0.04690 | 0.3580 | 661.1 | 428 | 1.248 | 0.04238 | 0.006496 | 1352 |
| C/T | 38 | 1.099 | 0.18057 |  |  | 59 | 0.9262 | 0.09369 |  |  | 97 | 0.994 | 0.09068 |  |  |
| rs2069747 |  |  |  |  |  |  |  |  |  |  |  |  |  |  |  |
| Codominant |  |  |  |  |  |  |  |  |  |  |  |  |  |  |  |
| C/C | 218 | 1.480 | 0.06777 | 0.2406 | 646.9 | 293 | 0.9942 | 0.04219 | 0.174 | 660 | 511 | 1.201 | 0.03913 | 0.9883 | 1359 |
| C/T | 9 | 1.059 | 0.33438 |  |  | 5 | 1.4348 | 0.34401 |  |  | 14 | 1.193 | 0.24440 |  |  |
| rs20541 |  |  |  |  |  |  |  |  |  |  |  |  |  |  |  |
| Codominant |  |  |  |  |  |  |  |  |  |  |  |  |  |  |  |
| G/G | 87 | 1.466 | 0.11163 | 0.002579 | 638.2 | 126 | 1.0672 | 0.05660 | 0.3457 | 661.8 | 213 | 1.230 | 0.05799 | 0.06704 | 1356 |
| A/G | 102 | 1.278 | 0.09783 |  |  | 129 | 0.9729 | 0.06889 |  |  | 231 | 1.108 | 0.05857 |  |  |
| A/A | 38 | 1.956 | 0.12586 |  |  | 43 | 0.8956 | 0.11917 |  |  | 81 | 1.393 | 0.10439 |  |  |
| Dominant |  |  |  |  |  |  |  |  |  |  |  |  |  |  |  |
| G/G | 87 | 1.466 | 0.11163 | 0.893481 | 648.3 | 126 | 1.0672 | 0.05660 | 0.1878 | 660.2 | 213 | 1.230 | 0.05799 | 0.55681 | 1359 |
| A/G-A/A | 140 | 1.462 | 0.08286 |  |  | 172 | 0.9536 | 0.05952 |  |  | 312 | 1.182 | 0.05154 |  |  |
| Recessive |  |  |  |  |  |  |  |  |  |  |  |  |  |  |  |
| A/G-G/G | 189 | 1.364 | 0.07379 | 0.001462 | 638.0 | 255 | 1.0195 | 0.04469 | 0.2923 | 660.8 | 444 | 1.166 | 0.04132 | 0.05813 | 1356 |
| A/A | 38 | 1.956 | 0.12586 |  |  | 43 | 0.8956 | 0.11917 |  |  | 81 | 1.393 | 0.10439 |  |  |
| Overdominant |  |  |  |  |  |  |  |  |  |  |  |  |  |  |  |
| G/G-A/A | 125 | 1.615 | 0.08867 | 0.012204 | 641.9 | 169 | 1.0235 | 0.05209 | 0.5709 | 661.6 | 294 | 1.275 | 0.05100 | 0.04984 | 1355 |
| A/G | 102 | 1.278 | 0.09783 |  |  | 129 | 0.9729 | 0.06889 |  |  | 231 | 1.108 | 0.05857 |  |  |
| rs1295685 |  |  |  |  |  |  |  |  |  |  |  |  |  |  |  |
| Codominant |  |  |  |  |  |  |  |  |  |  |  |  |  |  |  |
| G/G | 88 | 1.620 | 0.09530 | 0.007098 | 640.2 | 120 | 1.0402 | 0.06101 | 0.6629 | 663.1 | 208 | 1.286 | 0.05697 | 0.10187 | 1356 |
| A/G | 109 | 1.255 | 0.09877 |  |  | 136 | 0.9911 | 0.06631 |  |  | 245 | 1.109 | 0.05781 |  |  |
| A/A | 30 | 1.759 | 0.19470 |  |  | 42 | 0.9254 | 0.11108 |  |  | 72 | 1.273 | 0.11395 |  |  |
| Dominant |  |  |  |  |  |  |  |  |  |  |  |  |  |  |  |
| G/G | 88 | 1.620 | 0.09530 | 0.050615 | 644.4 | 120 | 1.0402 | 0.06101 | 0.4614 | 661.4 | 208 | 1.286 | 0.05697 | 0.08577 | 1356 |
| A/G-A/A | 139 | 1.364 | 0.08954 |  |  | 178 | 0.9756 | 0.05693 |  |  | 317 | 1.146 | 0.05169 |  |  |
| Recessive |  |  |  |  |  |  |  |  |  |  |  |  |  |  |  |
| G/G-A/G | 197 | 1.418 | 0.07031 | 0.086457 | 645.3 | 256 | 1.0141 | 0.04531 | 0.4553 | 661.4 | 453 | 1.190 | 0.04093 | 0.51817 | 1359 |
| A/A | 30 | 1.759 | 0.19470 |  |  | 42 | 0.9254 | 0.11108 |  |  | 72 | 1.273 | 0.11395 |  |  |
| Overdominant |  |  |  |  |  |  |  |  |  |  |  |  |  |  |  |
| G/G-A/A | 118 | 1.655 | 0.08635 | 0.002002 | 638.6 | 162 | 1.0104 | 0.05356 | 0.8383 | 661.9 | 280 | 1.282 | 0.05137 | 0.03298 | 1354 |
| A/G | 109 | 1.255 | 0.09877 |  |  | 136 | 0.9911 | 0.06631 |  |  | 245 | 1.109 | 0.05781 |  |  |
| rs848 |  |  |  |  |  |  |  |  |  |  |  |  |  |  |  |
| Codominant |  |  |  |  |  |  |  |  |  |  |  |  |  |  |  |
| C/C | 72 | 1.655 | 0.10815 | 0.031222 | 643.3 | 93 | 1.0321 | 0.07377 | 0.8883 | 663.7 | 165 | 1.304 | 0.06717 | 0.18066 | 1358 |
| A/C | 120 | 1.307 | 0.09231 |  |  | 147 | 0.9916 | 0.06077 |  |  | 267 | 1.133 | 0.05406 |  |  |
| A/A | 35 | 1.605 | 0.18129 |  |  | 58 | 0.9782 | 0.09465 |  |  | 93 | 1.214 | 0.09505 |  |  |
| Dominant |  |  |  |  |  |  |  |  |  |  |  |  |  |  |  |
| C/C | 72 | 1.655 | 0.10815 | 0.037124 | 643.9 | 93 | 1.0321 | 0.07377 | 0.6406 | 661.7 | 165 | 1.304 | 0.06717 | 0.08546 | 1356 |
| A/C-A/A | 155 | 1.374 | 0.08269 |  |  | 205 | 0.9878 | 0.05103 |  |  | 360 | 1.154 | 0.04698 |  |  |
| Recessive |  |  |  |  |  |  |  |  |  |  |  |  |  |  |  |
| C/C-A/C | 192 | 1.438 | 0.07139 | 0.354357 | 647.5 | 240 | 1.0073 | 0.04685 | 0.7738 | 661.8 | 432 | 1.198 | 0.04226 | 0.91401 | 1359 |
| A/A | 35 | 1.605 | 0.18129 |  |  | 58 | 0.9782 | 0.09465 |  |  | 93 | 1.214 | 0.09505 |  |  |
| Overdominant |  |  |  |  |  |  |  |  |  |  |  |  |  |  |  |
| C/C-A/A | 107 | 1.638 | 0.09341 | 0.008808 | 641.3 | 151 | 1.0114 | 0.05803 | 0.8372 | 661.9 | 258 | 1.271 | 0.05490 | 0.09312 | 1356 |
| A/C | 120 | 1.307 | 0.09231 |  |  | 147 | 0.9916 | 0.06077 |  |  | 267 | 1.133 | 0.05406 |  |  |
| rs2069750 |  |  |  |  |  |  |  |  |  |  |  |  |  |  |  |
| Codominant |  |  |  |  |  |  |  |  |  |  |  |  |  |  |  |
| G/G | 217 | 1.462 | 0.06887 | 0.7503 | 648.2 | 280 | 1.0188 | 0.04369 | 0.107 | 659.3 | 497 | 1.212 | 0.04005 | 0.287 | 1358 |
| C/G | 10 | 1.488 | 0.22411 |  |  | 18 | 0.7347 | 0.12901 |  |  | 28 | 1.004 | 0.13246 |  |  |
| rs847 |  |  |  |  |  |  |  |  |  |  |  |  |  |  |  |
| Codominant |  |  |  |  |  |  |  |  |  |  |  |  |  |  |  |
| C/C | 88 | 1.635 | 0.09408 | 0.010496 | 641.0 | 113 | 1.0304 | 0.06430 | 0.6869 | 663.2 | 201 | 1.295 | 0.05863 | 0.09651 | 1356 |
| T/C | 106 | 1.252 | 0.10150 |  |  | 140 | 1.0045 | 0.06270 |  |  | 246 | 1.111 | 0.05686 |  |  |
| T/T | 33 | 1.684 | 0.18335 |  |  | 45 | 0.9205 | 0.11534 |  |  | 78 | 1.244 | 0.11020 |  |  |
| Dominant |  |  |  |  |  |  |  |  |  |  |  |  |  |  |  |
| C/C | 88 | 1.635 | 0.09408 | 0.036051 | 643.9 | 113 | 1.0304 | 0.06430 | 0.5984 | 661.6 | 201 | 1.295 | 0.05863 | 0.05888 | 1356 |
| T/C-T/T | 139 | 1.355 | 0.08985 |  |  | 185 | 0.9841 | 0.05503 |  |  | 324 | 1.143 | 0.05069 |  |  |
| Recessive |  |  |  |  |  |  |  |  |  |  |  |  |  |  |  |
| C/C-T/C | 194 | 1.426 | 0.07114 | 0.178232 | 646.5 | 253 | 1.0160 | 0.04496 | 0.4097 | 661.2 | 447 | 1.194 | 0.04110 | 0.71044 | 1359 |
| T/T | 33 | 1.684 | 0.18335 |  |  | 45 | 0.9205 | 0.11534 |  |  | 78 | 1.244 | 0.11020 |  |  |
| Overdominant |  |  |  |  |  |  |  |  |  |  |  |  |  |  |  |
| C/C-T/T | 121 | 1.648 | 0.08436 | 0.002562 | 639.1 | 158 | 0.9991 | 0.05646 | 0.9371 | 661.9 | 279 | 1.281 | 0.05219 | 0.03522 | 1355 |
| T/C | 106 | 1.252 | 0.10150 |  |  | 140 | 1.0045 | 0.06270 |  |  | 246 | 1.111 | 0.05686 |  |  |
| **me:** mean in picogram per milliliters; **se:** standard error of mean; **p-value:** sex and age adjusted; **AIC:** Akaike Information Criterium for each inheritance models. p-value < 0.05 are considered significant. | | | | | | | | | | | | | | | |

| **Supplementary Table 6.** Correlations of Interleukin-13 circulating plasma concentrations with *IL13* SNVs in Patients with CL, Healthy Subjects, and Totals. | | | | | | | | | | | | | | | |
| --- | --- | --- | --- | --- | --- | --- | --- | --- | --- | --- | --- | --- | --- | --- | --- |
|  | Patients with CL | | | | | Healthy Subjects | | | | | Totals | | | | |
| rs1881457 | n | me | se | p-value | AIC | n | me | se | p-value | AIC | n | me | se | p-value | AIC |
| Codominant |  |  |  |  |  |  |  |  |  |  |  |  |  |  |  |
| A/A | 101 | 0.2162 | 0.10196 | 0.9117 | 493.6 | 162 | -0.8267 | 0.11231 | 0.8005 | 887.2 | 263 | -0.4262 | 0.08532 | 0.5912 | 1442 |
| A/C | 58 | 0.2760 | 0.14632 |  |  | 82 | -0.7305 | 0.14154 |  |  | 140 | -0.3135 | 0.11065 |  |  |
| C/C | 6 | 0.3480 | 0.35148 |  |  | 7 | -0.5496 | 0.66253 |  |  | 13 | -0.1353 | 0.39827 |  |  |
| Dominant |  |  |  |  |  |  |  |  |  |  |  |  |  |  |  |
| A/A | 101 | 0.2162 | 0.10196 | 0.6749 | 491.7 | 162 | -0.8267 | 0.11231 | 0.5573 | 885.3 | 263 | -0.4262 | 0.08532 | 0.3951 | 1441 |
| A/C-C/C | 64 | 0.2827 | 0.13595 |  |  | 89 | -0.7162 | 0.13918 |  |  | 153 | -0.2984 | 0.10642 |  |  |
| Recessive |  |  |  |  |  |  |  |  |  |  |  |  |  |  |  |
| A/A-A/C | 159 | 0.2380 | 0.08368 | 0.8435 | 491.8 | 244 | -0.7943 | 0.08832 | 0.6558 | 885.4 | 403 | -0.3870 | 0.06763 | 0.4471 | 1441 |
| C/C | 6 | 0.3480 | 0.35148 |  |  | 7 | -0.5496 | 0.66253 |  |  | 13 | -0.1353 | 0.39827 |  |  |
| Overdominant |  |  |  |  |  |  |  |  |  |  |  |  |  |  |  |
| A/A-C/C | 107 | 0.2236 | 0.09794 | 0.7251 | 491.7 | 169 | -0.8152 | 0.11070 | 0.6571 | 885.4 | 276 | -0.4125 | 0.08336 | 0.5559 | 1441 |
| A/C | 58 | 0.2760 | 0.14632 |  |  | 82 | -0.7305 | 0.14154 |  |  | 140 | -0.3135 | 0.11065 |  |  |
| rs1295687 |  |  |  |  |  |  |  |  |  |  |  |  |  |  |  |
| Codominant |  |  |  |  |  |  |  |  |  |  |  |  |  |  |  |
| C/C | 117 | 0.34366 | 0.09802 | 0.08653 | 488.8 | 160 | -0.8240 | 0.11129 | 0.8342 | 887.3 | 277 | -0.3308 | 0.08386 | 0.5532 | 1442 |
| C/G | 42 | 0.05659 | 0.15092 |  |  | 78 | -0.7352 | 0.15456 |  |  | 120 | -0.4581 | 0.11829 |  |  |
| G/G | 6 | -0.44160 | 0.39222 |  |  | 13 | -0.6517 | 0.37674 |  |  | 19 | -0.5854 | 0.28065 |  |  |
| Dominant |  |  |  |  |  |  |  |  |  |  |  |  |  |  |  |
| C/C | 117 | 0.34366 | 0.09802 | 0.05205 | 488.0 | 160 | -0.8240 | 0.11129 | 0.5673 | 885.3 | 277 | -0.3308 | 0.08386 | 0.2988 | 1440 |
| C/G-G/G | 48 | -0.00568 | 0.14145 |  |  | 91 | -0.7233 | 0.14224 |  |  | 139 | -0.4755 | 0.10879 |  |  |
| Recessive |  |  |  |  |  |  |  |  |  |  |  |  |  |  |  |
| C/C-C/G | 159 | 0.26783 | 0.08278 | 0.11087 | 489.2 | 238 | -0.7949 | 0.09021 | 0.7193 | 885.5 | 397 | -0.3693 | 0.06855 | 0.5282 | 1441 |
| G/G | 6 | -0.44160 | 0.39222 |  |  | 13 | -0.6517 | 0.37674 |  |  | 19 | -0.5854 | 0.28065 |  |  |
| Overdominant |  |  |  |  |  |  |  |  |  |  |  |  |  |  |  |
| C/C-G/G | 123 | 0.30536 | 0.09608 | 0.18157 | 490.0 | 173 | -0.8111 | 0.10652 | 0.6728 | 885.4 | 296 | -0.3472 | 0.08049 | 0.4290 | 1441 |
| C/G | 42 | 0.05659 | 0.15092 |  |  | 78 | -0.7352 | 0.15456 |  |  | 120 | -0.4581 | 0.11829 |  |  |
| rs2069744 |  |  |  |  |  |  |  |  |  |  |  |  |  |  |  |
| Codominant |  |  |  |  |  |  |  |  |  |  |  |  |  |  |  |
| C/C | 137 | 0.2598 | 0.09322 | 0.5675 | 491.5 | 197 | -0.8209 | 0.10393 | 0.4878 | 886.2 | 334 | -0.3776 | 0.07781 | 0.3276 | 1441 |
| C/T | 28 | 0.1551 | 0.15225 |  |  | 49 | -0.6086 | 0.14541 |  |  | 77 | -0.3309 | 0.11519 |  |  |
|  |  |  |  |  |  | 5 | -12.241 | 0.76992 |  |  | 5 | -12.241 | 0.76992 |  |  |
|  |  |  |  |  |  |  |  |  |  |  |  |  |  |  |  |
|  |  |  |  |  |  | 197 | -0.8209 | 0.10393 | 0.4539 | 885.1 | 334 | -0.3776 | 0.07781 | 0.8486 | 1442 |
|  |  |  |  |  |  | 54 | -0.6655 | 0.14873 |  |  | 82 | -0.3853 | 0.11849 |  |  |
|  |  |  |  |  |  |  |  |  |  |  |  |  |  |  |  |
|  |  |  |  |  |  | 246 | -0.7786 | 0.08817 | 0.4792 | 885.1 | 411 | -0.3689 | 0.06676 | 0.1367 | 1439 |
|  |  |  |  |  |  | 5 | -12.241 | 0.76992 |  |  | 5 | -12.241 | 0.76992 |  |  |
|  |  |  |  |  |  |  |  |  |  |  |  |  |  |  |  |
|  |  |  |  |  |  | 202 | -0.8309 | 0.10288 | 0.3055 | 884.6 | 339 | -0.3901 | 0.07753 | 0.8235 | 1442 |
|  |  |  |  |  |  | 49 | -0.6086 | 0.14541 |  |  | 77 | -0.3309 | 0.11519 |  |  |
| rs2069747 |  |  |  |  |  |  |  |  |  |  |  |  |  |  |  |
| Codominant |  |  |  |  |  |  |  |  |  |  |  |  |  |  |  |
| C/C | 160 | 0.2356 | 0.08376 | 0.6486 | 491.6 | 247 | -0.79940 | 0.08877 | 0.3015 | 884.5 | 407 | -0.3925 | 0.06788 | 0.2171 | 1440 |
| C/T | 5 | 0.4480 | 0.22204 |  |  | 4 | -0.05358 | 0.17229 |  |  | 9 | 0.2251 | 0.16250 |  |  |
| rs20541 |  |  |  |  |  |  |  |  |  |  |  |  |  |  |  |
| Codominant |  |  |  |  |  |  |  |  |  |  |  |  |  |  |  |
| G/G | 62 | 0.3659 | 0.13142 | 0.04101 | 487.2 | 103 | -0.7473 | 0.14824 | 0.7491 | 887.0 | 165 | -0.3290 | 0.11277 | 0.2580 | 1441 |
| A/G | 77 | 0.0372 | 0.11068 |  |  | 113 | -0.8583 | 0.12532 |  |  | 190 | -0.4954 | 0.09249 |  |  |
| A/A | 26 | 0.5534 | 0.23525 |  |  | 35 | -0.6773 | 0.20620 |  |  | 61 | -0.1528 | 0.17270 |  |  |
| Dominant |  |  |  |  |  |  |  |  |  |  |  |  |  |  |  |
| G/G | 62 | 0.3659 | 0.13142 | 0.22209 | 490.3 | 103 | -0.7473 | 0.14824 | 0.7166 | 885.5 | 165 | -0.3290 | 0.11277 | 0.5561 | 1441 |
| A/G-A/A | 103 | 0.1675 | 0.10364 |  |  | 148 | -0.8155 | 0.10725 |  |  | 251 | -0.4121 | 0.08198 |  |  |
| Recessive |  |  |  |  |  |  |  |  |  |  |  |  |  |  |  |
| A/G-G/G | 139 | 0.1838 | 0.08565 | 0.09093 | 488.9 | 216 | -0.8054 | 0.09626 | 0.6149 | 885.4 | 355 | -0.4181 | 0.07213 | 0.2121 | 1440 |
| A/A | 26 | 0.5534 | 0.23525 |  |  | 35 | -0.6773 | 0.20620 |  |  | 61 | -0.1528 | 0.17270 |  |  |
| Overdominant |  |  |  |  |  |  |  |  |  |  |  |  |  |  |  |
| G/G-A/A | 88 | 0.4213 | 0.11539 | 0.01600 | 485.9 | 138 | -0.7295 | 0.12204 | 0.4755 | 885.1 | 226 | -0.2814 | 0.09456 | 0.1426 | 1439 |
| A/G | 77 | 0.0372 | 0.11068 |  |  | 113 | -0.8583 | 0.12532 |  |  | 190 | -0.4954 | 0.09249 |  |  |
| rs1295685 |  |  |  |  |  |  |  |  |  |  |  |  |  |  |  |
| Codominant |  |  |  |  |  |  |  |  |  |  |  |  |  |  |  |
| G/G | 70 | 0.2300 | 0.12336 | 0.6822 | 493.0 | 101 | -0.7653 | 0.15615 | 0.9098 | 887.4 | 171 | -0.3579 | 0.11139 | 0.7709 | 1443 |
| A/G | 74 | 0.2006 | 0.11715 |  |  | 118 | -0.8253 | 0.11607 |  |  | 192 | -0.4299 | 0.09164 |  |  |
| A/A | 21 | 0.4284 | 0.27247 |  |  | 32 | -0.7184 | 0.22171 |  |  | 53 | -0.2640 | 0.18724 |  |  |
| Dominant |  |  |  |  |  |  |  |  |  |  |  |  |  |  |  |
| G/G | 70 | 0.2300 | 0.12336 | 0.9138 | 491.8 | 101 | -0.7653 | 0.15615 | 0.8465 | 885.6 | 171 | -0.3579 | 0.11139 | 0.7974 | 1442 |
| A/G-A/A | 95 | 0.2509 | 0.10903 |  |  | 150 | -0.8025 | 0.10255 |  |  | 245 | -0.3940 | 0.08239 |  |  |
| Recessive |  |  |  |  |  |  |  |  |  |  |  |  |  |  |  |
| G/G-A/G | 144 | 0.2149 | 0.08468 | 0.3909 | 491.1 | 219 | -0.7976 | 0.09517 | 0.7577 | 885.5 | 363 | -0.3960 | 0.07136 | 0.5771 | 1441 |
| A/A | 21 | 0.4284 | 0.27247 |  |  | 32 | -0.7184 | 0.22171 |  |  | 53 | -0.2640 | 0.18724 |  |  |
| Overdominant |  |  |  |  |  |  |  |  |  |  |  |  |  |  |  |
| G/G-A/A | 91 | 0.2758 | 0.11340 | 0.6410 | 491.6 | 133 | -0.7540 | 0.12964 | 0.6914 | 885.5 | 224 | -0.3357 | 0.09572 | 0.5313 | 1441 |
| A/G | 74 | 0.2006 | 0.11715 |  |  | 118 | -0.8253 | 0.11607 |  |  | 192 | -0.4299 | 0.09164 |  |  |
| rs848 |  |  |  |  |  |  |  |  |  |  |  |  |  |  |  |
| Codominant |  |  |  |  |  |  |  |  |  |  |  |  |  |  |  |
| C/C | 58 | 0.2540 | 0.13983 | 0.7868 | 493.3 | 81 | -0.7944 | 0.17888 | 0.5107 | 886.3 | 139 | -0.3569 | 0.12695 | 0.6947 | 1443 |
| A/C | 82 | 0.1990 | 0.10851 |  |  | 122 | -0.8618 | 0.12027 |  |  | 204 | -0.4354 | 0.09153 |  |  |
| A/A | 25 | 0.3553 | 0.24616 |  |  | 48 | -0.5873 | 0.16023 |  |  | 73 | -0.2645 | 0.14393 |  |  |
| Dominant |  |  |  |  |  |  |  |  |  |  |  |  |  |  |  |
| C/C | 58 | 0.2540 | 0.13983 | 0.8845 | 491.8 | 81 | -0.7944 | 0.17888 | 0.9495 | 885.6 | 139 | -0.3569 | 0.12695 | 0.8183 | 1442 |
| A/C-A/A | 107 | 0.2355 | 0.10071 |  |  | 170 | -0.7843 | 0.09767 |  |  | 277 | -0.3903 | 0.07735 |  |  |
| Recessive |  |  |  |  |  |  |  |  |  |  |  |  |  |  |  |
| C/C-A/C | 140 | 0.2218 | 0.08571 | 0.5477 | 491.5 | 203 | -0.8349 | 0.10134 | 0.2664 | 884.4 | 343 | -0.4036 | 0.07481 | 0.4827 | 1441 |
| A/A | 25 | 0.3553 | 0.24616 |  |  | 48 | -0.5873 | 0.16023 |  |  | 73 | -0.2645 | 0.14393 |  |  |
| Overdominant |  |  |  |  |  |  |  |  |  |  |  |  |  |  |  |
| C/C-A/A | 83 | 0.2845 | 0.12193 | 0.5684 | 491.5 | 129 | -0.7173 | 0.12706 | 0.4145 | 884.9 | 212 | -0.3251 | 0.09672 | 0.4524 | 1441 |
| A/C | 82 | 0.1990 | 0.10851 |  |  | 122 | -0.8618 | 0.12027 |  |  | 204 | -0.4354 | 0.09153 |  |  |
| rs2069750 |  |  |  |  |  |  |  |  |  |  |  |  |  |  |  |
| Codominant |  |  |  |  |  |  |  |  |  |  | 395 | -0.3792 | 0.06917 | 0.957 | 1442 |
| G/G | 159 | 0.25168 | 0.08353 | 0.5484 | 491.5 | 236 | -0.8042 | 0.09141 | 0.4273 | 885 | 21 | -0.3788 | 0.22912 |  |  |
| C/G | 6 | -0.01364 | 0.36546 |  |  | 15 | -0.5248 | 0.28387 |  |  |  |  |  |  |  |
| rs847 |  |  |  |  |  |  |  |  |  |  |  |  |  |  |  |
| Codominant |  |  |  |  |  |  |  |  |  |  |  |  |  |  |  |
| C/C | 71 | 0.2152 | 0.12251 | 0.7279 | 493.2 | 96 | -0.8082 | 0.16281 | 0.8098 | 887.2 | 167 | -0.3731 | 0.11381 | 0.6439 | 1443 |
| T/C | 71 | 0.2167 | 0.11790 |  |  | 121 | -0.8117 | 0.11347 |  |  | 192 | -0.4314 | 0.09095 |  |  |
| T/T | 23 | 0.4031 | 0.26417 |  |  | 34 | -0.6429 | 0.21500 |  |  | 57 | -0.2208 | 0.17892 |  |  |
| Dominant |  |  |  |  |  |  |  |  |  |  |  |  |  |  |  |
| C/C | 71 | 0.2152 | 0.12251 | 0.7857 | 491.8 | 96 | -0.8082 | 0.16281 | 0.8357 | 885.6 | 167 | -0.3731 | 0.11381 | 0.9200 | 1442 |
| T/C-T/T | 94 | 0.2623 | 0.10959 |  |  | 155 | -0.7747 | 0.10018 |  |  | 249 | -0.3832 | 0.08123 |  |  |
| Recessive |  |  |  |  |  |  |  |  |  |  |  |  |  |  |  |
| C/C-T/C | 142 | 0.2160 | 0.08471 | 0.4247 | 491.2 | 217 | -0.8102 | 0.09564 | 0.5156 | 885.2 | 359 | -0.4043 | 0.07181 | 0.3955 | 1441 |
| T/T | 23 | 0.4031 | 0.26417 |  |  | 34 | -0.6429 | 0.21500 |  |  | 57 | -0.2208 | 0.17892 |  |  |
| Overdominant |  |  |  |  |  |  |  |  |  |  |  |  |  |  |  |
| C/C-T/T | 94 | 0.2612 | 0.11244 | 0.7738 | 491.8 | 130 | -0.7650 | 0.13247 | 0.8074 | 885.6 | 224 | -0.3344 | 0.09620 | 0.4932 | 1441 |
| T/C | 71 | 0.2167 | 0.11790 |  |  | 121 | -0.8117 | 0.11347 |  |  | 192 | -0.4314 | 0.09095 |  |  |
| **me:** mean in picogram per milliliters; **se:** standard error of mean; **p-value:** sex and age adjusted; **AIC:** Akaike Information Criterium for each inheritance models. p-value < 0.05 are considered significant. | | | | | | | | | | | | | | | |

| **Supplementary Table** **7**. Haplotype analysis with the single nucleotide variants rs1295685, rs848 , and rs847 located in the 3’ untranslated region of *IL13* in Patients with CL (Cases) and healthy controls (HC). | | | | |
| --- | --- | --- | --- | --- |
| **Hap.** | **Cases** | **HC** | **p.value [OR (CI95%)]** | **Adj p.value [ORadj (CI95%)]** |
| AAC | 0.004 | 0.08 | 3.22e-28 [0.05 (0.03-0.11)] | 8.08e-27 [0.05 (0.02-0.11)] |
| GCT | 0.002 | 0.07 | 7.41-24 [0.04 (0.02-0.10)] | 2.69e-22 [0.04 (0.01-0.09)] |
| GAT | 0.01 | 0.02 | 0.032 [0.76 (0.45-1.28)] | 0.05 [0.76 (0.44-1.29)] |
| GAC | 0.04 | 0.05 | 0.487 [1.09 (0.79-1.51)] | 0.57 [1.02 (0.73-1.44)] |
| AAT | 0.35 | 0.29 | 0.0002 [1.43 (1.18-1.74)] | 0.0007 [1.32 (1.07-1.62)] |
| GCC | 0.57 | 0.48 | 3.94e-07 [1.67 (1.37-2.04)] | 5.55e-07 [1.55 (1.25-1.92)] |
